# IL‐33 regulates cytokine production and neutrophil recruitment via the p38 MAPK‐activated kinases MK2/3

**DOI:** 10.1111/imcb.12200

**Published:** 2018-10-19

**Authors:** Pierre C McCarthy, Iain R Phair, Corinna Greger, Katerina Pardali, Victoria A McGuire, Andrew R Clark, Matthias Gaestel, J Simon C Arthur

**Affiliations:** ^1^ Division of Cell Signalling and Immunology School of Life Sciences Wellcome Trust Building University of Dundee Dow St Dundee DD1 5EH UK; ^2^ MRC Protein Phosphorylation Unit School of Life Sciences Sir James Black Centre University of Dundee Dow St Dundee DD1 5EH UK; ^3^ Respiratory, Inflammation & Autoimmunity IMED Biotech Unit AstraZeneca Gothenburg, Mölndal 43183 Sweden; ^4^ Photobiology Unit Scottish Cutaneous Porphyria Service Ninewells Hospital and Medical School Dundee DD1 9SY UK; ^5^ Institute of Inflammation and Ageing College of Medical and Dental Sciences University of Birmingham Birmingham B15 2TT UK; ^6^ Institute for Cell Biochemistry Hannover Medical School Carl‐Neuberg‐Str. 1 Hannover 30623 Germany

**Keywords:** IL‐13, IL‐33, mast cell, MK2, Myd88, p38 MAPK

## Abstract

IL‐33 is an IL‐1‐related cytokine that can act as an alarmin when released from necrotic cells. Once released, it can target various immune cells including mast cells, innate lymphoid cells and T cells to elicit a Th2‐like immune response. We show here that bone marrow‐derived mast cells produce IL‐13, IL‐6, TNF, GM‐CSF, CCL3 and CCL4 in response to IL‐33 stimulation. Inhibition of the p38 MAPK, or inhibition or knockout of its downstream kinases MK2 and MK3, blocked the production of these cytokines in response to IL‐33. The mechanism downstream of MK2/3 was cytokine specific; however, MK2 and MK3 were able to regulate TNF and GM‐CSF mRNA stability. Previous studies in macrophages have shown that MK2 regulates mRNA stability via phosphorylation of the RNA‐binding protein TTP (Zfp36). The regulation of cytokine production in mast cells was, however, independent of TTP. MK2/3 were able to phosphorylate the TTP‐related protein Brf1 (Zfp36 l1) in IL‐33‐stimulated mast cells, suggesting a mechanism by which MK2/3 might control mRNA stability in these cells. In line with its ability to regulate *in vitro* IL‐33‐stimulated cytokine production, double knockout of MK2 and 3 in mice prevented neutrophil recruitment following intraperitoneal injection of IL‐33.

## Introduction

IL‐33 is an IL‐1‐related cytokine that was identified through screening for ligands for the IL‐1 receptor family member ST2,^1^ and it is now established as the major *in vivo* ligand for the ST2 receptor.^2^, ^3^, ^4^ Constitutive IL‐33 expression has been observed in non‐hematopoietic cells, primarily epithelial and endothelial cells. While IL‐1β and IL‐18 require cleavage by the inflammasome in order for their secretion and biological activity, this is not true for IL‐33. IL‐33 lacks a conventional signal peptide and caspase cleavage of IL‐33 results in its inactivation.^5^, ^6^ This led to the proposal that IL‐33 acts as an alarmin following its release from necrotic cells.^7^

The IL‐33 receptor comprises the ST2 (Il1rl1) chain in combination with the IL‐1RAcP protein.^8^ ST2 expression and IL‐33 responsiveness have been reported in a number of cells, notably mast cells,^9^ type 2 innate lymphoid cells^10^, ^11^, ^12^ and some Th subsets including Tregs and Th2 cells.^13^, ^14^, ^15^ Like other members of the IL‐1/TLR receptor superfamily, following ligand binding, the ST2/IL‐1RacP dimer is able to recruit the signaling adaptor Myd88.^16^, ^17^ Recruitment of Myd88 promotes the formation of a Myd88osome that includes IRAK4 as well as IRAK1 and/or IRAK2 that is able to activate Traf6.^18^ In agreement with this, IL‐33 requires Traf6 to activate both the MAPK and NF‐κB pathways,^19^ which in turn promote the production of proinflammatory mediators.^17^, ^20^, ^21^ For example, IL‐33‐stimulated mast cells have been shown to secrete IL‐6, IL‐13, TNF, MCP‐1 and prostaglandin D2.^16^, ^22^, ^23^, ^24^ In contrast to IgE receptor‐mediated mast cell activation, IL‐33 stimulation alone does not promote mast cell degranulation.^1^, ^16^

The p38 MAPK family consists of four isoforms and acts downstream of cellular stress and inflammatory signals. A role for p38 in the regulation of cytokine production was initially suggested by the finding that a class of pyridinyl imidazoles typified by SB203580, reduced TNF production via inhibition of p38. This led to the development of a large number of p38 inhibitors, most of which target the p38α and β isoforms, although work with gene targeted mice has shown that in macrophages p38α, and not β, is the critical isoform for the regulation of TLR‐induced proinflammatory cytokine production.^18^ p38α is able to activate further downstream kinases, including MKs and MSKs, which can contribute to the ability of p38 to regulate cytokine production.^18^ While MK2 and MK3 are solely activated by p38 *in vivo*, MSK1 and the related kinase MSK2 are direct substrates for both p38α and ERK1/2.^18^ Work in macrophages has shown that MSKs induce anti‐inflammatory feedback pathways and are required for the production of IL‐10 by these cells.^25^, ^26^ Knockout of MK2 was found to reduce TNF production in response to TLR agonists both *in vivo* and in isolated macrophages.^27^ While MK2 appears to be the more dominant isoform, some compensation does exist between MK2 and MK3, as double knockout of both MK2 and MK3 resulted in a greater suppression of TNF production than knockout of MK2 alone following intraperitoneal injection of LPS in mice.^28^ In macrophages, the major mechanism by which MK2 and MK3 regulate the production of TNF is via phosphorylation of the mRNA‐binding protein TTP (also known as Zfp36).^29^, ^30^ TTP is an mRNA‐binding protein that recognizes AU‐rich elements in the 3′UTR of certain mRNAs including that of TNF.^31^ Once bound, TTP can both inhibit the translation of the mRNA and promote its degradation. TTP is phosphorylated by MK2 on at least two sites and this inhibits the ability of TTP to repress translation or promote RNA degradation.^30^, ^32^, ^33^ A critical role for TTP in repressing TNF production has been shown both *in vivo* and in isolated macrophages using TTP knockout mice.^34^, ^35^ Surprisingly, bone marrow‐derived mast cells from TTP knockout mice showed normal production of TNF and IL‐6 in response to LPS.^21^ This was attributed to a low basal expression of TTP in mast cells as judged by immunoblotting.^21^ Despite this, TTP may still play a role in mast cells under some circumstances; mast cells upregulate TTP mRNA in response to IL‐4 stimulation and this has been proposed to explain the repression of IgE‐induced TNF production by IL‐4.^36^ Recently, MK2 and MK3 have been suggested to play a role in IL‐6 and IL‐13 induction in IL‐33‐stimulated mast cells and IL‐13 in dendritic cells; however, the substrate targeted by MK2 in these cells is not clear.^24^, ^37^ We show here that knockout or inhibition of MK2 and MK3 in mast cells blocks TNF, IL‐6, IL‐13, GM‐CSF, CCL3 and CCL4 production in response to IL‐33, and that this occurs via both transcriptional and post‐transcriptional mechanisms. We also show that the TTP‐related protein Brf1 is expressed in mast cells and is phosphorylated by MK2/3 in these cells following IL‐33 stimulation.

## Results

### IL‐33 induced cytokine production in BMMCs requires p38 MAPK

**Figure 1:**
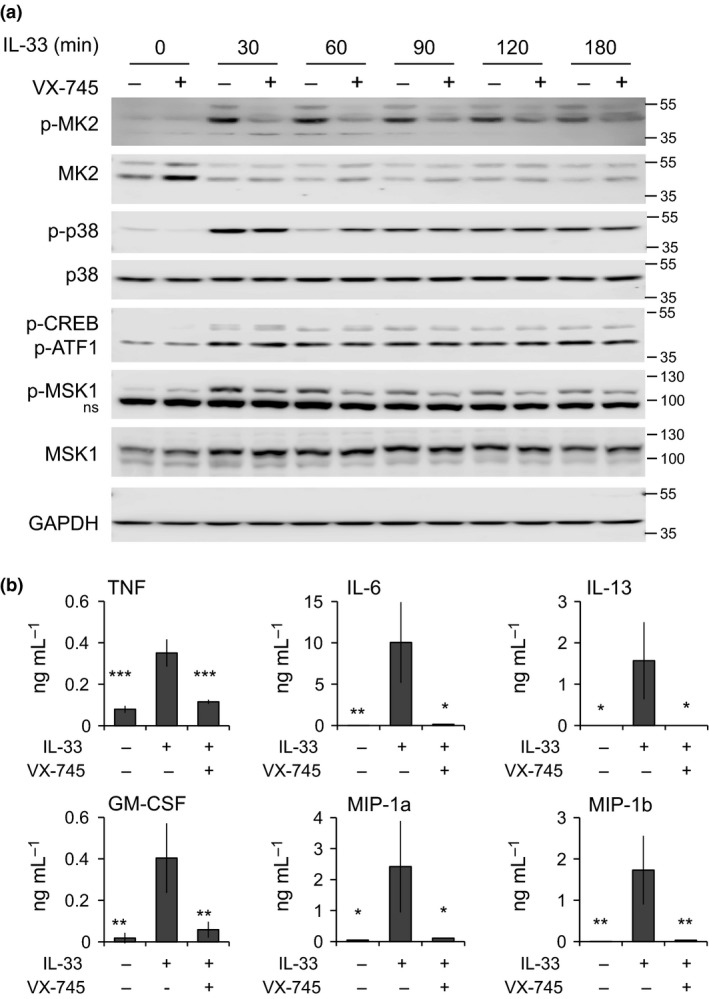
p38 is required for cytokine secretion in IL‐33‐stimulated BMMCs. **(a)** BMMCs were isolated from wild‐type mice. Where indicated, cells were pretreated for 1 h with 1 μm VX‐745 before stimulation with 10 ng mL ^−1^ IL‐33 for the indicated times. The levels of the indicated proteins were then determined by immunoblotting. **(b)** As in **a,** but BMMCs were stimulated with 10 ng mL^−1^ IL‐33 for 8 h and the levels of TNF, IL‐6, IL‐13, GM‐CSF, CCL3 and CCL4 determined as described in the methods. Graphs represent the mean and standard deviation of results from independent cultures from four mice. For a comparison with the IL‐33‐stimulated condition, a *P*‐value (two‐tailed Student's *t*‐test) of <0.05 is indicated by *, <0.01 by ** and <0.001 by ***. BMMC, bone marrow‐derived mast cells.

The IL‐33 receptor is part of IL‐1 receptor/TLR family of receptor family and acts via Myd88 to stimulate MAPK and NF‐κB activation. Consistent with previous reports,^38^, ^39^, ^40^ IL‐33 was found to activate p38α in bone marrow‐derived mast cells (BMMCs), as judged by phosphorylation of its TXY activation motif. The activation of p38α was absent in BMMCs from Myd88 knockout mice (Supplementary figure 1). To examine the roles of p38α in IL‐33‐induced cytokine induction, BMMCs were treated with the p38α/β inhibitor VX‐745 prior to stimulation with IL‐33. Pretreatment with VX‐745 did not affect the phosphorylation of p38α on its TXY motif, which is catalyzed by the upstream kinases MKK3 and ‐6, but did block the phosphorylation of the p38α substrate MK2 (Figure 1a). IL‐33 also induced the activation of MSK1, as judged by its phosphorylation on Ser376, a site that correlates to MSK1 activation,^41^ as well as the phosphorylation of the MSK substrate CREB. VX‐745 did not prevent MSK1 or CREB phosphorylation, suggesting that as in other cell types, IL‐33 activates MSKs in part via the ERK1/2 pathway. To examine the role of p38α in cytokine induction in BMMCs, cells were stimulated for 8 h with IL‐33 and the secretion of TNF, IL‐6, IL‐13 and GM‐CSF, as well as the chemokines CCL3 and CCL4, was analyzed. IL‐33 was able to stimulate the production of the cytokines and chemokines tested, and in each case, pretreatment with VX‐745 was able to inhibit their production (Figure 1b).

### IL‐33‐induced cytokine production requires the p38‐activated kinases MK2 and MK3

As p38α can mediate its effects via downstream kinases, the role of MSK1/2 or MK2/3 knockout in IL‐33‐induced cytokine induction was examined. Bone marrow from MSK1/2 double knockout mice was able to differentiate into BMMCs, as judged by similar expression of c‐kit and FcεRI and ST2 to wild‐type BMMCs (Supplementary figure 2). IL‐33 induced phosphorylation of MSK1 and its substrates CREB and ATF1 (Supplementary figure 3a, b). In line with the ability of both p38 and ERK1/2 to activate MSK1, inhibition of both pathways was required to fully block MSK1 and CREB phosphorylation (Supplementary figure 3a). As expected, IL‐33‐stimulated phosphorylation of CREB was lost in MSK1/2 knockout cells (Supplementary figure 3b). MSK1/2 knockout cells produced similar levels of TNF, IL‐6, IL‐13 and GM‐CSF to wild‐type cells in response to IL‐33 stimulation (Supplementary figure 3c). In line with the levels of secreted cytokines, the induction of TNF, IL‐6, IL‐13 and GM‐CSF mRNA was similar in wild‐type and MSK1/2 knockout BMMCs. Although MSK1/2 knockout did not have a major impact on IL‐33‐induced cytokine induction, it did greatly reduce the induction of nur77 mRNA (Supplementary figure 3d), an immediate early gene previously shown to be both MSK and CREB dependent in fibroblasts.^42^, ^43^

**Figure 2:**
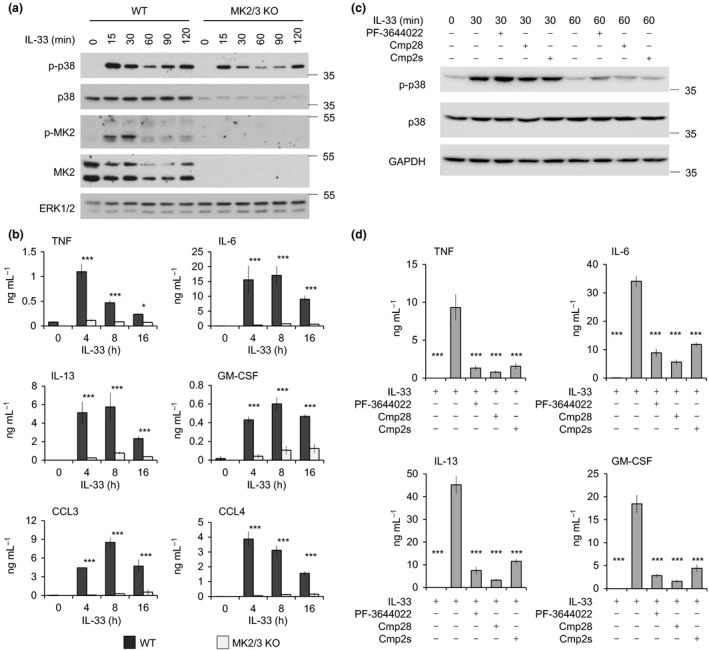
Regulation of IL‐33‐induced cytokine production by MK2/3. **(a)** Wild‐type or MK2/3 knockout BMMCs were stimulated for the indicated times with 10 ng mL^−1^ IL‐33 and the levels of total and phospho‐p38, total and phospho‐MK2 determined by immunoblotting. Total ERK1/2 was examined as a loading control. **(b)** BMMCs from wild‐type or MK2/3 knockout mice were stimulated with 10 ng mL^−1^ IL‐33 for 0, 4, 8 or 16 h, and the levels of TNF, IL‐6, IL‐13, GM‐CSF, CCL3 and CCL4 secreted into the media were determined. Graphs represent the mean and standard deviation of results from independent cultures from four wild‐type mice or three MK2/3 knockout mice. Two‐way ANOVA indicated a significant effect of genotype on all the cytokines tested, (*P* < 0.001, *F* = 233, 135, 102, 424, 334 or 514 for TNF, IL‐6, IL‐13, GM‐CSF, CCL3 or CCL4, respectively). For individual time points, *P*‐value (post hoc Holm–Sidak testing) of <0.05 is indicated by *, <0.01 by ** and <0.001 by ***. N.D. indicates a condition for which values not determined. **(c)** Wild‐type BMMCs were pretreated with 5 μm PF‐3644022, 5 μm Cmp28 or 5 μm Cmp2s for 1 h where indicated and then stimulated with 10 ng mL^−1^ IL‐33 for the indicated times. The levels of the indicated proteins were determined by immunoblotting. **(d)** BMMCs were pretreated with 5 μm PF‐3644022, 5 μm Cmp28 or 5 μm Cmp2s for 1 h as shown and then either left unstimulated or stimulated with 10 ng mL^−1^ IL‐33 for 8 h. The levels of TNF, IL‐6, IL‐13 and GM‐CSF secreted into the media were determined as described in the methods. Graphs represent the mean and standard deviation of results from four independent stimulations. For a comparison with the no inhibitor, IL‐33 stimulated condition, a *P*‐value (post hoc Holm–Sidak test after one‐way ANOVA) of <0.001 is indicated by ***. BMMC, bone marrow‐derived mast cells.

MK2/3 knockout did not affect the differentiation of BMMCs, and both wild‐type and MK2/3 knockout BMMCs expressed similar levels ST2 (Supplementary figure 2). Knockout of MK2/3 in BMMCs resulted in a decreased expression of p38α (Figure 2a). This decreased level of p38α is due to a kinase activity‐independent role for MK2 in stabilizing p38 prior to activation, and is consistent with what has been reported in other cell types in MK2 single or MK2/3 double knockouts.^27^, ^44^, ^45^ Despite this, p38α activation, as judged by its phosphorylation, following IL‐33 stimulation was still apparent, albeit at a lower level than in wild‐type cells (Figure 2a). MK2/3 knockout BMMCs produced greatly reduced levels of TNF, IL‐6, IL‐13, GM‐CSF, CCL4 and CCL4 following IL‐33 stimulation relative to wild‐type cells (Figure 2b). While these data are consistent with a role for MK2 and MK3 in directly regulating cytokine production in BMMCs, the results in the knockout could also be explained by an indirect mechanism because of the decreased levels of p38α in the MK2/3 knockout cells. To resolve this, three different MK2/3 inhibitors were used, PF‐3644022, Cmp28 and Cmp2s. MK2 and MK3 are known to phosphorylate Hsp27.^46^ As Hsp27 is not detectable in BMMCs, Cmp28 and Cmp2s were titrated in HeLa cells to determine the concentration required to block Hsp27 phosphorylation in response to anisomycin, a strong activator of the p38–MK2 pathway.^47^ This showed that 5 μm of either compound was able to inhibit MK2/3 in cells (Supplementary figure 4). Previous studies have shown that 5 μm of PF‐3644022 is able to block Hsp27 phosphorylation in HeLa cells.^48^ To examine potential off‐target activities of Cmp28 and Cmp2s, the compounds were screened *in vitro* against a panel of 290 kinases. At 1 μm, both these compounds inhibited MK2 and MK3 by more than 90%. Cmp2s inhibited PRAK by 72%, but none of the other kinases were inhibited by more than 50%. Cmp28 did not inhibit any of the other kinases tested by more than 25% (Supplementary figure 4, table 1). The screening of PF‐3644022 showed that it was more potent against MK2 than MK3, and that it showed more off‐target activities than Cmp2s and Cmp28 (Supplementary figure 4, table 1).

As the stabilization of p38α by MK2 is independent of the kinase activity of MK2,^45^ inhibitors of MK2 and MK3 would not be expected to affect p38α expression or activation. Consistent with this, treatment of BMMCs with PF‐3644022, Cmp28 or Cmp2s did not affect p38 levels or activation in response to IL‐33 in BMMCs (Figure 2c). Pretreatment of BMMCs with PF‐3644022, Cmp2s or Cmp28 for 1 h before stimulation with IL‐33 inhibited the production of TNF, IL‐6, IL‐13 and GM‐CSF (Figure 2d). Together, these results are consistent with MK2 and MK3 directly regulating cytokine induction downstream of p38α in IL‐33‐stimulated mast cells. Similar to that observed in BMMCs, cultured peritoneal mast cells also secreted TNF, IL‐6, IL‐13 and GM‐CSF in response to IL‐33, and this was blocked by the MK2 inhibitor Cmp2s (Supplementary figure 5).

The role of MK2 and MK3 has previously been examined in TLR signaling which, similar to IL‐33, can act via Myd88‐dependent signaling. This work has focused predominantly in macrophages and dendritic cells where MK2 is required for maximal TNF production.^28^ It is less clear if loss of MK2 and MK3 results in a general block in cytokine induction in these cells as was seen in BMMCs (Figure 2). To determine whether this was due to a difference between IL‐33 and TLR agonists or a mast cell‐specific effect, cytokine induction in LPS‐stimulated MK2/3 knockout mast cells and bone marrow‐derived macrophages (BMDMs) was examined. LPS was used, as IL‐33 did not induce cytokine production in BMDMs (data not shown). BMDMs produced TNF, IL‐6, IL‐12p70 and IL‐12p40 in response to LPS. MK2/3 knockout BMDMs produced lower amounts of TNF than wild‐type cells in response to LPS; however, IL‐6, IL‐12p70 and IL‐12p40 production was not reduced (Supplementary figure 6a). BMDMs were obtained via culture of bone marrow cells in M‐CSF. Differentiation of bone marrow cells with GM‐CSF gives rise to a heterogeneous macrophage/dendritic cell population.^49^ LPS‐induced TNF secretion was decreased in MK2/3 knockout cells relative to wild‐type cells in GM‐CSF differentiated cultures while only minor effects were seen on IL‐6, IL‐13 and IL‐12p40 secretion (Supplementary figure 6b). To examine the role of MK2/3 in cytokine production in TLR‐stimulated mast cells, BMMCs were stimulated with TLR4 agonist LPS. Similar to IL‐33, LPS was able to stimulate TNF, IL‐6, IL‐13 and GM‐CSF production in BMMCs, and in each case, the production of these cytokines was reduced by MK2/3 knockout (Supplementary figure 6c). In line with this, LPS‐induced cytokine production in BMMCs was inhibited by the MK2/3 inhibitors PF‐3604422 and Cmp2s (Supplementary figure 7).

**Figure 3:**
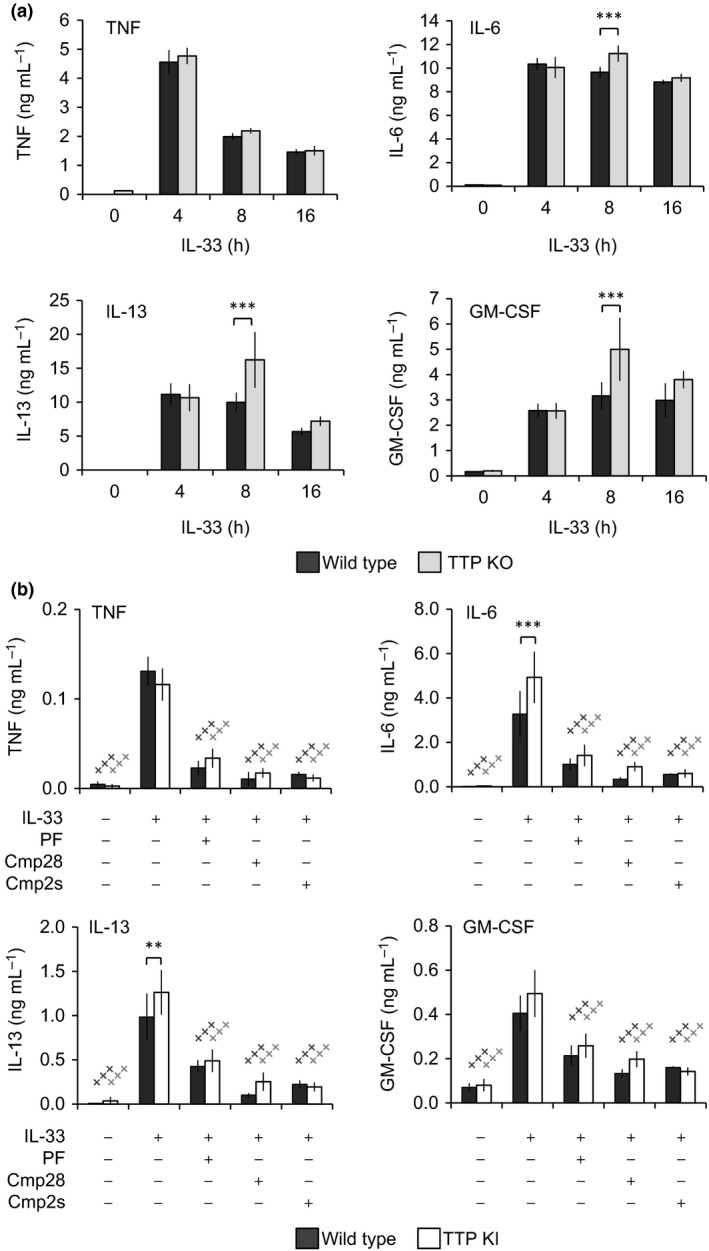
IL‐33‐induced cytokine production is independent of TTP in mast cells. **(a)** BMMCs were cultured from wild‐type or TTP knockout mice. Cells were stimulated for 0, 4, 8 or 16 h with 10 ng mL^−1^ IL‐33 and levels TNF, IL‐6, IL‐13 and GM‐CSF secreted into the media measured. **(b)** BMMCs were cultured from wild‐type or TTP knockin mice in which the MK2 phosphorylation sites in TTP were mutated to alanine. Where indicated cells were pretreated with 5 μm PF‐3644022, 5 μm Cmp28 or 5 μm Cmp2s for 1 h. Cells were then either left unstimulated of stimulated with 10 ng mL^−1^ IL‐33 for 8 h. TNF IL‐6, IL‐13 and GM‐CSF secretion was then measured. In **a** and **b**, graphs show the mean and standard deviation of independent cultures from four mice per genotype. A *P*‐value (Holm–Sidak test following two‐way ANOVA testing) between wild type and knockout of <0.05 is indicated * and *P* < 0.001 by ***. A *P*‐value for the comparison of inhibitor treated conditions and the IL‐33‐stimulated sample within one genotype of <0.001 is shown by +++.

In macrophages, MK2 regulates TNF production via the phosphorylation of TTP, a protein that binds to AU‐rich elements in the 3′UTR of a subset on mRNAs resulting in destabilization of the mRNA.^31^, ^50^ To determine whether MK2/3‐mediated TTP phosphorylation could explain the effects of MK2/3 knockout in mast cells, BMMCs were derived from TTP knockout bone marrow. Loss of TTP did not have a major effect on the IL‐33 induced secretion of TNF, IL‐6, IL‐13 or GM‐CSF (Figure 3a). To confirm these findings, BMMCs were also generated from mice carrying alanine mutations at the two major MK2 phosphorylation sites, Ser52 and Ser178, in TTP.^30^ Again, the IL‐33 induced secretion of TNF, IL‐6, IL‐13 and GM‐CSF was similar in TTP knockin and wild‐type BMMCs. Furthermore, pretreatment of the knockin cells with either PF‐3604422, Cmp2s or Cmp28 was able to inhibit cytokine induction, indicating that MK2 and MK3 could act independently of TTP phosphorylation (Figure 3b).

### The p38–MK2/3 pathway regulates IL‐33 induced translation of TNF

**Figure 4:**
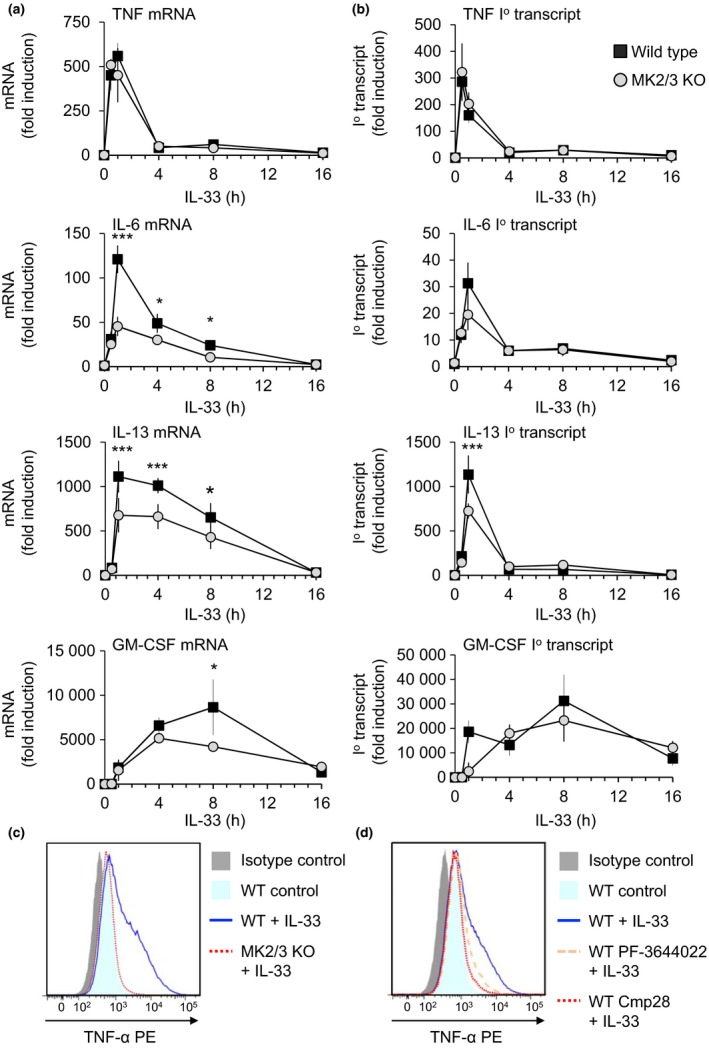
Effect of MK2/3 knockout in BMMCs on IL‐33‐induced cytokine mRNA levels. **(a)** BMMCs from wild‐type or MK2/3 knockout mice were stimulated with 10 ng mL^−1^ IL‐33 for the indicated times. Cells were then lysed, total RNA was isolated and cytokine induction was determined by qPCR as described in the methods. The induction of TNF, IL‐6, IL‐13 and GM‐CSF mRNA relative to wild‐type unstimulated cells is shown. Two‐way ANOVA indicated a significant effect of genotype on IL‐6 (*F* = 55.12, *P* < 0.001), IL‐13 (*F* = 23.27, *P* < 0.001) and GM‐CSF (*F* = 6.23, *P* = 0.019). **(b)** As in **a**, but primary unspliced transcript (1^o^ transcript) levels in the same cDNA samples were determined. In **a** and **b**, graphs represent the mean and standard deviation of results from independent cultures from four wild‐type mice or three MK2/3 knockout mice. Two‐way ANOVA indicated a significant effect of genotype on IL‐6 (*F* = 6.92, *P* = 0.014). In (a) and (b) for comparisons between genotype at individual time points, a *P*‐value (post hoc Holm–Sidak test following two‐way ANOVA) of <0.05 is indicated by *, <0.01 by ** and <0.001 by ***. **(c)** Wild‐type or MK2/3 knockout BMMCs were stimulated for 4 h with 10 ng mL^−1^ in the presence of 3 μg mL^−1^ Brefeldin A and 2 μm Monensin to block cytokine secretion. Cells were then fixed and permeabilized, stained for TNF and analyzed by flow cytometry. **(d)** As in **c**, except wild‐type cells were pretreated with 5 μm PF‐3644022 for 1 h where indicated. In **c** and **d**, data are representative of two biological replicates.

To examine the mechanism by which MK2/3 regulates IL‐33‐induced cytokine production, the effects of MK2/3 knockout on cytokine mRNA induction was examined. IL‐33 stimulation increased the mRNA levels for TNF, IL‐6, GM‐CSF and IL‐13 in wild‐type BMMCs. Loss of MK2 and MK3 did not greatly affect the induction of either TNF mRNA or the unspliced primary TNF transcripts relative to wild‐type cells (Figure 4a, b). To examine the upregulation of TNF protein, cells were stimulated with IL‐33 in the presence of agents that block secretion and then intracellular levels of TNF determined by flow cytometry. This showed that, unlike wild‐type cells, MK2/3 knockout BMMC failed to upregulate TNF protein in response to IL‐33 (Figure 4c). Similar effects on TNF protein induction were seen by pretreating wild‐type cells with the MK2 inhibitor PF‐3644022 (Figure 4d). MK2/3 knockout BMMCs showed reduced mRNA induction of IL‐6 and IL‐13 in response to IL‐33 relative to wild‐type cells, with the maximal effect seen around 1 h of stimulation. This correlated to a lower induction of the primary transcript for IL‐13, and to a lesser extent IL‐6, in MK2/3 knockout cells at this time point (Figure 4a, b). For GM‐CSF, the initial induction of mRNA was normal in the MK2/3 knockouts; however, levels were lower in MK2/3 knockouts compared to wild‐type BMMCs at 8 h of LPS stimulation (Figure 4a).

**Figure 5:**
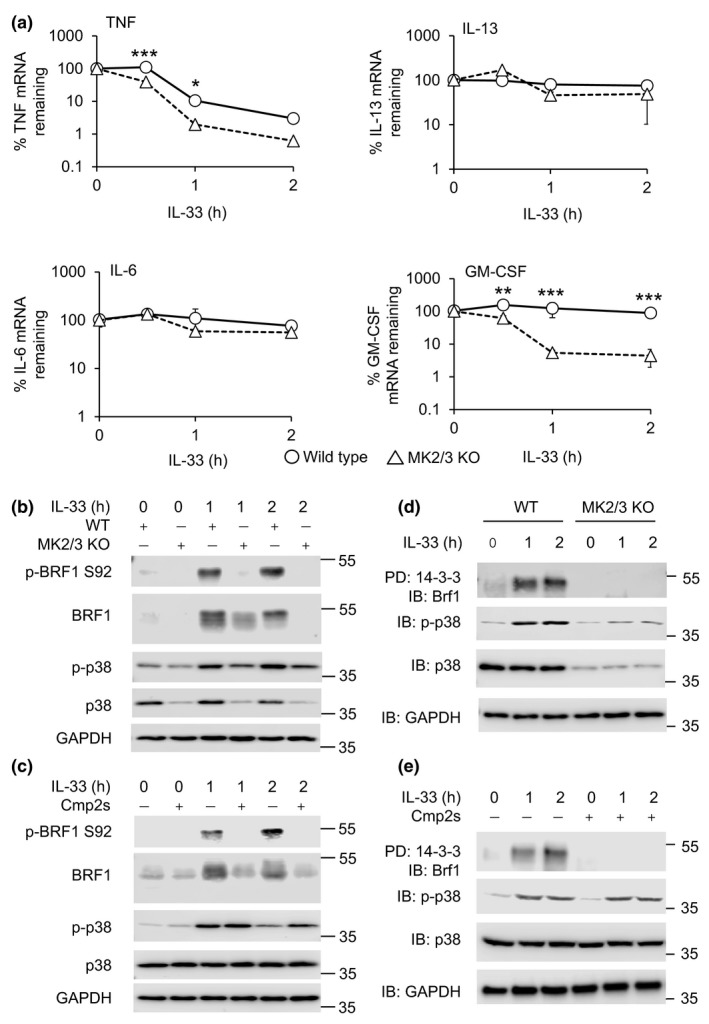
Effect of MK2/3 knockout on cytokine mRNA stability in IL‐33‐stimulated BMMCs. **(a)** Wild‐type or MK2/3 knockout BMMCs were stimulated with 10 ng mL^−1^ IL‐33 for 1 h. A quantity of 5 μg mL^−1^ actinomycin D and 50 μm DRB were then added to block transcription. Total RNA was isolated at 0, 0.5, 1 and 2 h after addition of actinomycin D and DRB and the levels of TNF, IL‐6, IL‐13 and GM‐CSF mRNA determined by qPCR. Graphs show the mean and standard deviation of independent cultures from four mice per genotype. Two‐way ANOVA showed a significant effect of genotype for TNF and GM‐CSF (*F* = 112.255, *P* < 0.001 and *F* = 51.582 *P* < 0.001, respectively). For individual time points, in post hoc testing *P*‐values between wild type and knockout of <0.05 are indicated * and <0.001 by ***. **(b)** Wild‐type or MK2/3 KO BMMCs were stimulated with 10 ng mL^−1^ IL‐33 for the indicated times. Cells were then lysed and the levels phosho‐Ser92 Brf1, Brf1, p‐p38, total p38 and GAPDH were determined by immunoblotting. **(c)** Wild‐type BMMCs were incubated where indicated with 5 μm VX‐745 or 5 μm Cmp28. Cells were stimulated with 10 ng mL^−1^ IL‐33 for the times shown and blotted for the indicated proteins. **(d)** Wild‐type or MK2/3 knockout BMMCs were stimulated with 10 ng mL^−1^ IL‐33 for the indicated times. Cells were then lysed and 14‐3‐3 pulldowns performed as described in the methods. Pulldowns were blotted for the presence of Brf1 while lysates were blotted for phospho p38, total p38 and GAPDH. **(e)** Wild‐type BMMCs where incubated for 1 h with DMSO or 5 μm Cmp2s before stimulation with 10 ng mL^−1^ IL‐33 for the indicated times. Cells were then lysed and 14‐3‐3 pulldowns performed as described in the methods. Pulldowns were blotted for the presence of Brf1 while lysates were blotted for phospho p38, total p38 and GAPDH. DRB 5,6‐dichloro‐1*β*‐1‐ribofuranosylbenzimidazole.

To determine whether MK2/3 knockout might affect cytokine RNA stability, cells were stimulated for 1 h with IL‐33 and further transcription blocked by the addition of DRB (5,6‐dichloro‐1*β*‐1‐ribofuranosylbenzimidazole) and actinomycin D and the decay of mRNA levels measured over time. TNF mRNA was unstable and decayed with a half‐life of approximately 55 min in wild‐type cells (Figure 5a). In MK2/3 knockout BMMCs, TNF mRNA was less stable with a half‐life of approximately 30 min. In wild‐type cells, IL‐6, IL‐13 and GM‐CSF mRNAs were found to be stable (half‐life >2 h). Knockout of MK2/3 did not have a major effect on the stability of IL‐6 or IL‐13 mRNA; however, MK2/3 knockout did decrease the stability of the GM‐CSF mRNA (Figure 5a). Similar results were observed when cells were stimulated for 3 h with IL‐33 before the addition of DRB and actinomycin D (data not shown).

### MK2/3 phosphorylate Brf1 in response to IL‐33

While the effects of MK2/3 on TNF and GM‐CSF production BMMCs are TTP independent, MK2 has been reported to phosphorylate Brf1, a TTP‐related protein, *in vitro* and in the fibrosarcoma cell line, HT1080.^51^ Brf1 protein levels were increased in IL‐33 stimulated BMMCs (Figure 5a), as were mRNA levels for both Brf1 and Brf2 (Supplementary figure 8). Multiple bands were observed in the immunoblots, consistent with the multiple phosphorylation sites known to occur on Brf1 affecting its electrophoretic mobility on SDS‐polyacrylamide gels. Knockout of MK2 and MK3 resulted in a decreased phosphorylation of Brf1 as judged by the decrease in the upper band in the Brf1 blots from MK2/3 knockout compared to wild‐type BMMCs. Similar results were observed when cells were treated with the MK2/3 inhibitor cmp2s (Figure 5c). Brf1 has been reported to be phosphorylated on several sites including Ser92. Blotting with a phospho‐specific antibody for this site showed that MK2/3 knockout or MK2/3 inhibitor reduced the IL‐33 induced phosphorylation of Brf1 on this site in mast cells (Figure 5b, c). Phosphorylation of Brf1 has been reported to enable it to interact with 14‐3‐3 proteins.^52^, ^53^ In line with this, more Brf1 was observed in 14‐3‐3 pulldowns from lysates from IL‐33‐stimulated BMMCs relative to unstimulated cells. This was blocked by knockout of MK2/3 (Figure 5d) or pretreatment of wild‐type cells with an MK2/3 inhibitor (Figure 5e). To confirm that the Brf1 in the 14‐3‐3 pulldowns was phosphorylated, 14‐3‐3 pulldowns were treated with λ phosphatase and then analyzed by immunoblotting. This showed that the Brf1 in the phosphatase‐treated samples ran at a lower molecular weight, as would be expected with the removal of phosphate groups (Supplementary figure 9). Blotting of the pulldowns with an antibody that recognizes the phosphorylation of Brf1 on Ser92 also indicated that Brf1 was phosphorylated in the pulldowns and that this phosphorylation can be removed by the phosphatase treatment (Supplementary figure 9).

### PI3K signaling is required for maximal IL‐33‐induced cytokine production

**Figure 6:**
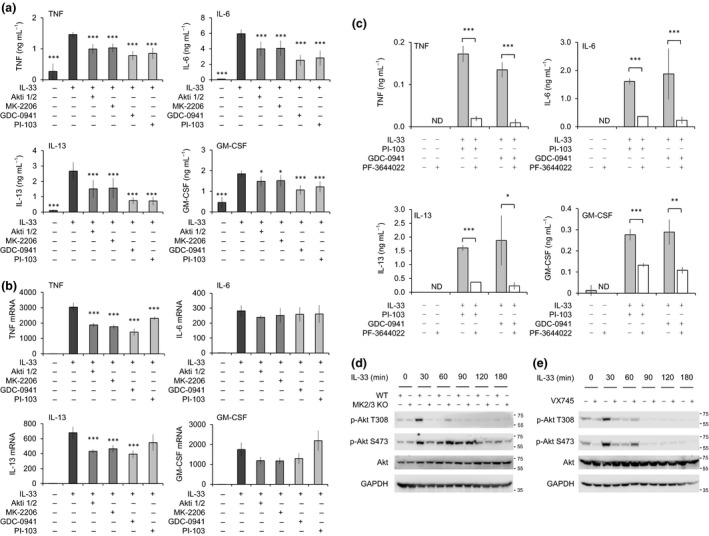
Inhibitors of the PI3 kinase–Akt pathway reduce IL‐33‐induced cytokine induction. **(a)** Wild‐type BMMCs were pretreated where indicated with 1 μm PI‐103, 1 μm GDC‐094, 1 μm Akti 1/2 or 1 μm MK2206. Cells were then stimulated with 10 ng mL^−1^ IL‐33 for 8 h or left unstimulated and the levels of TNF, IL‐6, IL‐13 and GM‐CSF secreted into the media determined. **(b)** As (a) except cells were stimulated for 1 h with 10 ng mL^−1^ IL‐33. Total RNA was then extracted and the induction of TNF, IL‐6, IL‐13 and GM‐CSF mRNA determined relative to unstimulated cells. In (a) and (b), a *P* < 0.5 is indicated by * and *P* < 0001 by *** (Holm–Sidak test following one‐way ANOVA). **(c)** Wild‐type BMMCs were pretreated where indicated with 1 μm PI‐103, 1 μm GDC‐094 or 5 μm PF‐3604422. Cells were then stimulated with 10 ng mL^−1^ IL‐33 for 8 h or left unstimulated and the levels of TNF, IL‐6, IL‐13 and GM‐CSF secreted into the media determined. In (a–c), graphs show the mean and standard deviation of independent cultures from four mice. A *P*‐value (two‐tailed Student's *t*‐test) between the IL‐33‐stimulated cells and other conditions of <0.05 is indicated *, <0.01 by ** and <0.001 by ***. **(d)** Wild‐type MK2/3 KO BMMCs were stimulated with 10 ng mL^−1^ IL‐33 for the indicated times. Cells were then lysed and the levels phospho‐Ser473 Akt, phospho‐Thr308 Akt, total Akt and GAPDH were determined by immunoblotting. **(e)** Wild‐type BMMCs were incubated where indicated with 1 μm VX‐745. Cells were stimulated with 10 ng mL^−1^ IL‐33 for the times shown and blotted for the indicated proteins.

In addition to being phosphorylated by MK2 and/or 3, Brf1 has also been reported as a substrate for Akt.^52^, ^53^ A role for MK2 and 3 in the activation of Akt has previously been found in TLR‐stimulated macrophages.^48^ To determine whether Akt might play a role in regulating IL‐33‐induced cytokine production, the Akt inhibitors Akti 1/2 and MK‐2206 as well as the PI3 kinase inhibitors GDC‐0941 and PI‐103 were used. All four inhibitors reduced the induction of TNF, IL‐6, IL‐13 and GM‐CSF (Figure 6a). Analysis of cytokine mRNA induction showed that inhibition of PI3 kinase or Akt reduced the induction of TNF and IL‐13 mRNA in response to IL‐33 (Figure 6b). GM‐CSF mRNA was not significantly affected (*P *> 0.05) by PI3 kinase inhibitors, although some reduction was seen in the presence of Akt inhibitors. IL‐6 mRNA levels were not affected by either PI3 kinase or Akt inhibitors following 1 h of IL‐33 stimulation (Figure 6b). While both MK2/3 and Akt are involved in IL‐33‐induced cytokine induction, the smaller inhibition seen by blocking PI3 kinase compared to MK2/3 indicate that the effects of MK2/3 knockout cannot be fully explained by cross talk between the MK2/3 and Akt pathways. To confirm this, MK2/3 inhibitors were added in combination with a PI3 kinase inhibitor. As in Figure 6a, GDC‐0941 and PI‐103 reduced IL‐33‐induced TNF, IL‐6, IL‐13 and GM‐CSF production. The remaining cytokine production in the presence of PI3 kinase inhibitors could however be further reduced by the addition of the MK2 inhibitor PF‐3604422 (Figure 6c). Finally, we tested if the phosphorylation of Akt on Thr308 and Ser473 required MK2/3 in IL‐33‐stimulated BMMCs. Both residues were phosphorylated in response to IL‐33 in wild‐type cells. In MK2/3 knockout cells, the phosphorylation of Akt on both Thr308 and Ser473 was reduced following 30 min of stimulation; however, at 60 min after IL‐33 stimulation, Akt phosphorylation on Ser473 was slightly enhanced in the MK2/3 knockout relative to wild‐type cells (Figure 6d). Similar effects were observed with the p38 inhibitor VX‐745 (Figure 6e). This would indicate that p38–MK2/3 signaling regulates the kinetics of Akt activation, but is not completely essential for IL‐33‐induced Akt activation.

### MK2 and MK3 regulate IL‐33‐induced neutrophil recruitment *in vivo*

**Figure 7:**
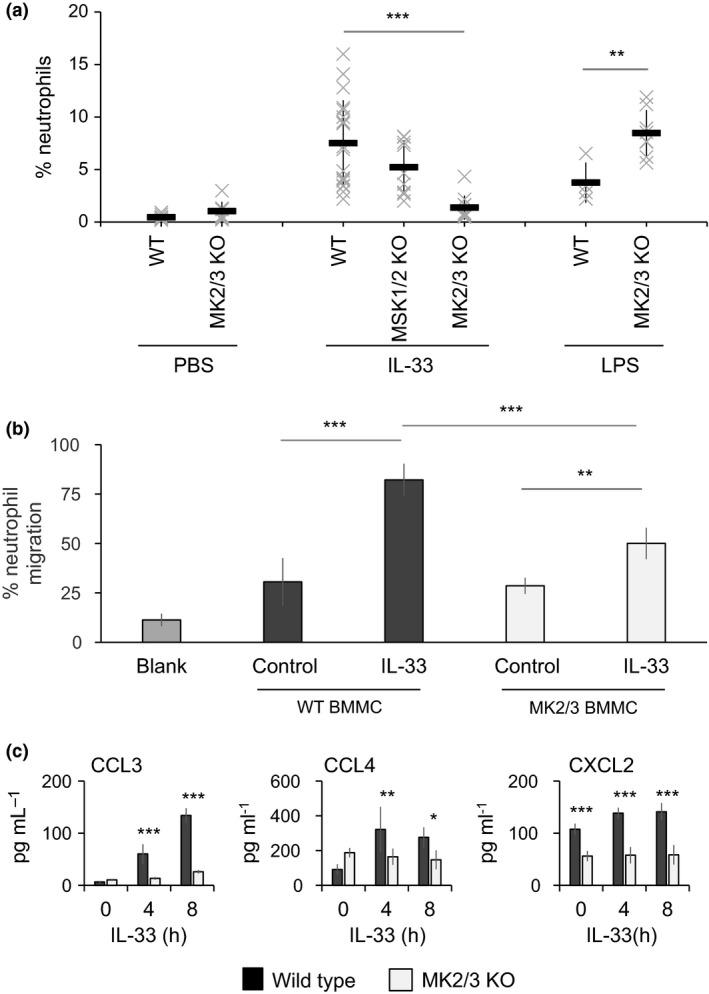
MK2/3 are required for neutrophil recruitment following intraperitoneal injection of IL‐33. **(a)** Wild type, MK2/3 or MSK1/2 were injected with either PBS, 5 μg kg^−1^ IL‐33 or 2 mg kg^−1^ LPS. After 3 h, mice were sacrificed and the peritoneum washed to obtain cells. The % of neutrophils (CD11b^+^/Gr1^+^) in the peritoneal wash was determined by staining for CD11b and Gr1 and analyzed by flow cytometry. Graphs show average and standard deviation with crosses representing data for an individual mouse. Two‐way ANOVA testing between wild‐type and MK2/3 knockout mice indicated a significant interaction between genotype and treatment (*F* = 19.34, *P* < 0.001). For post hoc analysis on the effect of genotype within treatment groups, a *P* < 0.01 is indicated ** and I < 0.001 by ***. The difference between MSK1/2 knockout and wild‐type animals treated with IL‐33 was not significant (*P *< 0.05, Student's *t*‐test). **(b)** Transwell assays were used to determine the migration of wild‐type neutrophils in response to wild‐type or MK2/3 knockout peritoneal mast cells (4 biological replicates per genotype) that were either unstimulated or treated with IL‐33, as described in the methods. Blank indicated wells with neutrophils but no mast cells. Two‐way ANOVA testing between wild‐type and MK2/3 knockout mast cells indicated a significant interaction between genotype and treatment (*F* = 12.404, *P* < 0.004). For post hoc analysis on the effect of genotype within treatment groups, a *P* < 0.01 is indicated by ** and *P* < 0.001 by ***. **(c)** Peritoneal mast cells were stimulated with 10 ng mL^−1^ IL‐33 for the indicated times and the levels of CCL3, CCL4 and CXCL2 in the media determined. Data show mean and standard deviation of four biological replicates per genotype. Two‐way ANOVA testing between wild‐type and MK2/3 knockout mast cells indicated a significant effect of genotype (*F* = 160.7, *P* < 0.001 for CCL3; *F* = 5.26, *P *= 0.034 for CCL2 and *F* = 151.7, *P *< 0.001 for CXCL2). For post hoc analysis on the effect of genotype at specific times, a *P* < 0.05 in indicated by *, <0.01 by ** and <0.001 by ***.

Intraperitoneal injection of IL‐33 in mice has previously been shown to promote neutrophil recruitment in wild‐type but not W^sh/sh^ mice, which lack mature mast cells.^54^ We therefore tested the effect of IL‐33 injection in MK2/3 double knockout mice (Figure 7a). Control experiments using LPS demonstrated that MK2/3 knockout mice recruited more neutrophils compared to wild‐type animals, arguing against an essential role for MK2/3 in neutrophil recruitment. In contrast, IL‐33 injection recruited much fewer neutrophils in MK2/3 knockout mice relative to wild‐type controls (Figure 7a). Knockout of MSK1 and 2 did not affect IL‐33‐induced neutrophil recruitment in this model (Figure 7a) consistent with the minor effects of MSKs on IL‐33‐induced cytokine production in isolated mast cells (Supplementary figure 2). To confirm that loss of MK2/3 in mast cells was able to affect the ability of these cells to secrete chemoattractant for neutrophils, peritoneal mast cells were cultured from wild‐type and MK2/3 knockout mice. These cells were then used in Transwell assays to examine their ability to promote the migration of wild‐type neutrophils. The presence of mast cells in the lower chamber was able to stimulate a low level of neutrophil migration. Stimulation of the wild‐type mast cells with IL‐33 resulted in an increased neutrophil migration. IL‐33 stimulation of MK2/3 knockout cells resulted in an increase in neutrophil migration, but this was lower than seen with wild‐type mast cells (Figure 7b). IL‐33 stimulated the production of the chemokines CCL3 and CCL4 by peritoneal mast cells, and this was reduced by knockout of MK2 and 3 (Figure 7c). CCL3 has previously been reported to be a relatively poor chemoattractant for neutrophils, but its ability to promote neutrophil recruitment is increased when the neutrophils are stimulated with GM‐CSF.^55^ In line with this, we observed a greater attraction of neutrophils to a combination of CCL3 and GM‐CSF than CCL3 alone in Transwell assays (Supplementary figure 10). CXCL2 was also detected in the media from peritoneal mast cells. While the levels were not increased by IL‐33 treatment, lower levels were detected from MK2/3 knockout than wild‐type mast cells (Figure 7c).

## Discussion

p38α has an established role in regulating cytokine production downstream of TLR signaling in macrophages and dendritic cells. With the exception of TLR3, TLRs can stimulate p38 activity via Myd88, a pathway shared with IL‐1 receptor family members including the ST2/IL‐1RAcP IL‐33 receptor. We show here that treatment of BMMCs with the p38α/β inhibitor VX‐745 greatly inhibited the IL‐33 induced production of TNF, IL‐6, IL‐13, GM‐CSF, CCL3 and CCL4. This is in agreement with previous studies have also shown that p38 inhibitors can block IL‐33‐induced TNF, IL‐6 and IL‐13 production in BMMCs.^38^, ^39^ p38α regulates cell function in part via the activation of two groups of downstream kinases, MK2/3 and MSK1/2, both of which impact on TLR‐induced cytokine production in macrophages.^18^ Both MK2/3 and MSK1/2 were activated in response to IL‐33 in BMMCs. In the case of MSK1/2, inhibition of both the p38α and ERK1/2 MAPK pathways was required to block IL‐33‐induced MSK1 activation and phosphorylation of the MSK substrate CREB, a finding consistent with MSK activation in response to TLR‐stimulated macrophages.^25^ MSK1/2 knockout did not have a major effect on cytokine production in IL‐33‐stimulated BMMCs. While MSKs are known to regulate Myd88‐dependent cytokine induction in macrophages and dendritic cells, they are not required for TLR‐induced IL‐10 production in B cells.^25^, ^56^, ^57^ The role of MSKs in cytokine induction therefore seems to be cell type specific.

In contrast to MSK1/2, MK2/3 knockout had a much greater impact on cytokine production in IL‐33‐stimulated BMMCs. MK2/3 knockout greatly reduced TNF, IL‐6, IL‐13, GM‐CSF, CCL3 and CCL4 secretion in response to IL‐33 in BMMCs, a finding consistent with a recent report showing MK2/3 knockout reduced IL‐33 stimulated IL‐6 and IL‐13 production in BMMCs.^40^ MK2/3 knockout reduces p38α levels because of a noncatalytic role for MK2 in stabilizing p38α. The results from the MK2/3 knockout cells could be interpreted as either being due to a loss of p38α or a direct role for MK2/3 catalytic activity in regulating cytokine induction. We therefore examined the effects of three MK2/3 inhibitors—these did not affect p38α expression levels but did replicate the effects of MK2/3 knockout on cytokine production.

In both macrophages and dendritic cells, MK2 is required for maximal TNF production;^28^ however, in these cell types, MK2 and 3 are not essential for the production of all cytokines. For example, MK2 knockout reduced IL‐6 production in macrophages on prolonged LPS stimulation but initial IL‐6 production was not reduced,^58^ while in dendritic cells MK2 knockout did not reduce IL‐6 or IL‐12 production.^59^ In agreement with these reports, we found that MK2/3 knockout did not prevent LPS‐induced IL‐6, IL‐13 or IL‐12p40 production in BMDMs or GM‐CSF differentiated bone marrow cells. The reason for the more extensive role of MK2 and MK3 in mast cells is not clear; however, our data would suggest that MK2 and MK3 regulate cytokines in mast cells via different mechanisms than they use in macrophages.

The regulation of cytokine production by MK2 and MK3 in mast cells is likely to involve regulation of both transcription and mRNA stability, and relative contribution may be cytokine specific. For both IL‐6 and IL‐13, MK2/3 knockout reduced the induction of the primary transcript, suggesting a role for MK2/3 in the transcription of these genes. In contrast, for TNF and GM‐CSF MK2/3 knockout did not have a negative effect on induction of the primary transcript but did affect the stability of the mRNA suggesting that MK2/3 regulate these genes via a post‐transcriptional mechanism. The post‐transcriptional regulation of cytokines by MK2/3 in macrophages has been shown to involve the protein TTP, which regulates stability and/or translation of AU‐rich element containing mRNAs, including TNF.^40^ TTP, however, did not contribute to the regulation of cytokine production in IL‐33‐stimulated BMMCs. Neither TTP knockout nor Ser to Ala knockin mutation of the two major MK2 phosphorylation sites in TTP affected IL‐33 stimulated production of TNF, IL‐6, IL‐13 or GM‐CSF in BMMCs. MK2/3 did, however, regulate mRNA stability in IL‐33‐stimulated BMMCs; TNF and GM‐CSF mRNA stability were reduced in MK2/3 knockout BMMCs.

Brf1 (Zfp36 l1) and Brf2 (Zfp36 l2) are related to TTP, and similar to TTP, are known to regulate mRNA stability.^26^, ^50^, ^52^, ^53^ The role of Brf1 and 2 in immunity, however, has not been studied as extensively as that of TTP. Brf1 and Brf2 are involved in early B‐cell development where they regulate senescence to enable recombination at the immunoglobulin locus^60^ while in T‐cell development they are involved in the β‐selection checkpoint, and loss of Brf1 and ‐2 results in leukemia.^61^ The roles of Brf1 and ‐2 in innate immune cells are not well understood. Brf1 can be induced by inflammatory stimuli in macrophages; however, Brf1 knockout, unlike TTP knockout, does not affect TNF or IL‐6 induction in these cells.^62^ Immunoblotting showed that BMMCs expressed Brf1 and that MK2/3 knockout or inhibition reduced Brf1 phosphorylation on Ser92. Two additional sites exist in Brf1 that conform to the MK2 consensus sequence, and it is possible that these sites are also phosphorylated by MK2/3 in IL‐33‐stimulated mast cells. The lack of phospho‐specific antibodies to these sites means we were not able to address this directly. The MK2 consensus sequence (ϕ‐X‐R‐X‐X‐pS/T) is similar to that required for binding to 14‐3‐3 proteins. Following IL‐33 stimulation, Brf1 was able to bind to 14‐3‐3 proteins and this was blocked by MK2/3 inhibitors or MK2/3 knockout. It is therefore possible that MK2/3 regulate cytokine stability downstream of IL‐33 via Brf1 phosphorylation resulting in Brf1 being sequestered by 14‐3‐3 proteins.

In addition to regulating TTP in TLR‐stimulated macrophages, MK2/3 also contribute to the activation of the PI3 kinase–Akt pathway. While the molecular mechanism behind this is unclear, in macrophages MK2/3 were found to act at the level of PIP3 production in the membrane, either via promoting PI3 kinase activity or inhibition of PIP3 phosphatases.^48^ We found that in BMMCs, IL‐33 was able to promote activation of Akt as judged by phosphorylation of Akt on Ser473 and Thr308. Drube *et al*. have also recently reported that IL‐33 can induce Ser473 phosphorylation in Akt, although Thr308, which is essential for Akt activation, was not addressed in this study.^40^ Drube *et al*. also reported that MK2/3 knockout abolished IL‐33‐induced Akt Ser473 phosphorylation and suggested that this contributed to the ability of MK2 and MK3 to regulate IL‐6 and IL‐13 production. In contrast, we did not see complete inhibition of Ser473 or Thr308 phosphorylation by MK2/3 knockout or p38 inhibition, although MK2/3 were more important in regulating the kinetics of Akt phosphorylation in response to IL‐33, with the initial phosphorylation of Akt being slower in the MK2/3 knockouts. The reason for this difference is not clear. Interestingly, the remaining cytokine induction in the presence of PI3 kinase or Akt inhibitors was still sensitive to MK2 inhibitors, indicating that in our experiments MK2/3 could regulate cytokine induction via a PI3 kinase–Akt independent mechanism.

MK2/3 knockout reduced neutrophil recruitment to the peritoneal cavity following intraperitoneal injection of IL‐33. Previous studies using this model have found that neutrophil recruitment did not occur in W^sh^/W^sh^ mice, which lack mast cells. This could be rescued by injection into the peritoneal cavity of wild‐type BMMCs but not BMMCs deficient in the IL‐33 receptor.^63^ *In vivo*, neutrophil recruitment is tightly regulated and may involve waves of different factors, including CXCL1/2, CCL3/4 and leukotriene B4 (LTB4).^64^, ^65^ The major chemoattractant for neutrophils in the intraperitoneal IL‐33 model is not known. In response to intraperitoneal injection of LPS, neutrophil recruitment can be reduced by antibodies blocking CXCL1 or CXCL2; however, a combination of both antibodies did not completely block neutrophil recruitment consistent with the involvement of other factors.^63^ Neutralizing antibodies against CXCL1 alone had no effect on neutrophil recruitment following intraperitoneal injection of IL‐33.^54^ LPS could activate both macrophages and mast cells in the peritoneal cavity, and thus both cells types could contribute to neutrophil recruitment in this model. Of note, MK2/3 knockout did not decrease neutrophil recruitment in response to LPS, which is consistent with the reduced dependence on MK2 and MK3 for the production of proinflammatory cytokines in macrophages relative to mast cells. In contrast, mast cells comprise the major ST2^+^ cell population in the peritoneal wash. The low numbers of mast cells present, however, makes direct measurement of chemokines in the peritoneal wash following IL‐33 injection problematic and we failed to detect cytokines in the peritoneal wash following IL‐33 injection, most probably due to the dilution during the washing procedure. In BMMC cultures, IL‐33 strongly induced the mRNA for CCL3 and CCL4. mRNA for CXCL1 and CXCL2 was also induced, but the fold induction and relative level of these mRNAs was less than for CCL3 and CCL4. Isolated peritoneal mast cells produced CCL3 and CCL4 in response to IL‐33, and this was reduced in MK2/3 knockouts relative to wild‐type mast cells. CXCL2 was also detected in the media, but little stimulation was seen in response to IL‐33. As peritoneal mast cells did not produce high levels of the classical neutrophil chemokines CXCL1 and ‐2, it is possible that neutrophil recruitment in this model may require the production of multiple factors. TNF knockout BMMCs were reported to be less effective than wild‐type BMMCs at rescuing neutrophil recruitment in the W^sh^/W^sh^ mice.^54^ GM‐CSF, another MK2/3‐dependent cytokine produced by mast cells, may also play a role in priming neutrophils; while CCL3 is a poor chemoattractant for naïve neutrophils *in vitro*, GM‐CSF is reported to prime neutrophils to make them sensitive to CCL3 as a chemoattractant.^55^ Further studies will be required to address these questions. Our data would also not exclude a role for MK2/3 in other cell types *in vivo*. Type II innate lymphoid cells also express the IL‐33 receptor and are present in the mesenteric membranes.^13^ It is possible that IL‐33 also induced chemokines in these cells *in vivo* and that this could contribute to the neutrophil recruitment.

Together these studies show that MK2 and MK3 are critical for regulating cytokine production in IL‐33‐ or LPS‐stimulated mast cells. The way in which MK2/3 regulate these processes is cytokine specific and may occur via transcriptional or post‐transcriptional mechanisms depending on the cytokine. The roles of MK2 and MK3 in IL‐33‐stimulated mast cells appear more extensive than those in TLR‐stimulated macrophages. IL‐33 also acts on ILC2s and T‐cell subsets, it will therefore be interesting to determine how MK2 and 3 affect IL‐33 responses in these cells.

## Methods

### Mice

TTP knockout, TTP S52A/S178A double knockin, MK2/3 double knockout, MSK1/2 double knockout and Myd88 knockout mice have been described previously and had been backcrossed onto C57Bl/6 for at least 12 generations.^27^, ^30^, ^34^, ^44^, ^47^, ^66^ Mice were housed in individually ventilated cages and allowed free access to food and water. Colonies were maintained under specific pathogen‐free conditions and work was approved via local ethical review and carried out subject to a UK Home Office license.

### Cell culture

BMMCs were derived from adult mice. Mice were sacrificed and femurs excised under sterile conditions. The bone marrow was flushed from the femurs with sterile PBS and the bone marrow suspension was passed through a 100‐μm cell strainer (Greiner bio‐one, Stonehouse, UK). Cells were centrifuged at 340 *g* for 5 min, and the pellet was resuspended in 25 mL mast cell media [RPMI 1640 medium containing 10% FBS (Biosera/Labtech, Heathfield, UK), 5 mm l‐Glutamine (GIBCO Life Technologies), 100 U mL^−1^ Penicillin (GIBCO Life Technologies), 100 μg mL^−1^ Streptomycin (GIBCO Life Technologies, Fisher Scientific, Loughborough, UK), 25 mm HEPES (Lonza, Edinburgh, UK), 1 mm sodium pyruvate (Lonza), 1X nonessential amino acids (Lonza), 50 μm 2‐mercaptoethanol and 30 ng mL^−1^ IL‐3 (PeproTech. London, UK)] and transferred to a 75‐cm^2^ tissue culture flask. The cells in suspension were cultured for 5–6 weeks at 37°C and 5% CO_2_, and were split twice a week to refresh the media and maintain their density at approximately 1 × 10^6^ cells mL^−1^. For measurement of cytokine production, cells were suspended in fresh media and then rested for 48 h before stimulation with IL‐33.

To culture peritoneal mast cells from mice, the peritoneal cavity was washed with PBS with 2 mm EDTA and 0.5% BSA. Cells were pelleted by centrifugation and resuspended in RPMI 1640 supplemented with 10% FBS (Biosera/Labtech), 5 mm l‐Glutamine (GIBCO Life Technologies), 100 U mL^−1^ Penicillin (GIBCO Life Technologies), 100 μg mL^−1^ Streptomycin (GIBCO Life Technologies), 25 mm HEPES (Lonza), 1 mm sodium pyruvate (Lonza), 1X nonessential amino acids (Lonza), 50 μm 2‐mercaptoethanol, 20 ng mL^−1^ SCF (PeproTech) and 30 ng mL^−1^ IL‐3 (PeproTech) and incubated at 37°C and 5% CO_2_. After 24 h, non‐adherent cells were discarded and the media replaced. Thereafter, both suspension and adherent cells were passaged into fresh media every 3 days and the cells used for experiments at 14 days. Analysis of the cells by flow cytometry showed that at 14 days they were >94% positive of c‐kit and FcεR and expressed the ST2 receptor.

BMDMs were cultured as described previously while GM‐CSF bone marrow‐differentiated cells were cultured as for BMDMs except that the M‐CSF in the culture media was replaced with 5 ng mL^−1^ GM‐CSF (PeproTech).^67^

Unless otherwise indicated, kinase inhibitors were dissolved in DMSO and used at the following final concentrations: VX‐745 (Selleck, Stratech, Ely, UK), 1 μm; PI‐103 (Merck Millipore, Watford, UK), 1 μm; GDC‐0941 (Axon Medichem, Groningen, Netherlands), 1 μm; Akti 1/2 (Merck Millipore), 1 μm; MK2206 (Selleck), 1 μm; PF‐364402 (Tocris, Bio‐Techne, Abingdon, UK), 5 μm; Cmp28 (compound 28 in Ref. 68, 69, synthesized in house), 5 μm; Cmp2s (Compound 2s in Ref. 68, 69 synthesized in house), 5 μm. Synthesis of Cmp28 and Cmp2s was based on previous publications.^68^, ^69^ *In vitro* kinase screening data for Cmp28 and Cmp2s were carried out by Merck Millipore and for PF 364402 by the MRC International Centre for Kinase Profiling (Dundee, http://www.kinase-screen.mrc.ac.uk). Cells were stimulated as indicated in the legends using 10 ng mL^−1^ murine IL‐33 (PeproTech) or 100 ng mL^−1^ LPS (*Escherichia coli* strain O26:B6; Sigma; L2654). For the zero‐time stimulations in the figures, cells did not receive any IL‐33 or LPS. The inhibitors did not compromise the viability of the mast cells at the concentrations and times used in this study (Supplementary figure 11). IL‐33 and GM‐CSF were from PeproTech and CCL3 was from Biolegend UK (London).

### Flow cytometry of mast cells

Cells were pelleted and resuspended in PBS containing 1% FCS and incubated with FcBlock (1:50, BD Biosciences, San Jose, US) for 10 min and then stained with anti‐c‐Kit‐FITC (1 in 500, BD Biosciences, #553354), anti‐FcεR1‐PE (1 in 400, eBiosciences VWR International Ltd Lutterworth, UK, #12‐5898‐81) and anti‐ST2‐BV421 (1 in 400, BioLegend #145309) for 30 min. Cells were then washed twice and analyzed by flow cytometry. For analysis, live cells were identified by gating based on forward and side scatter.

### Immunoblotting

Cells were lysed in triton lysis buffer: 50 mm Tris‐HCl (pH 7.5), 1 mm EGTA, 1 mm EDTA, 1 mm sodium orthovanadate, 50 mm sodium fluoride, 1 mm sodium pyrophosphate, 0.27 m sucrose, 1% (vol/vol) Triton X‐100, 0.1% (vol/vol) 2‐mercaptoethanol, 1 μg mL^−1^ aprotinin, 1 μg mL^−1^ leupeptin, 1 mm PMSF. Lysates were clarified by centrifugation (20 800 *g* for 10 min at 4°C) and supernatants snap‐frozen and stored at −80°C. Protein concentration was determined with Coomassie Protein Assay Reagent (Fisher Scientific, Loughborough, UK). Proteins were separated on 10% polyacrylamide gels and immunoblotting carried out using standard techniques. Antibodies recognizing phospho‐Thr180/Tyr182 p38 (#4511), total p38 (#9212), total ERK1/2 (#9102), phospho‐Ser473 Akt (#9271), phospho‐Thr308 Akt (#9275), BRF1/2 (#2119), phospho‐Ser376 MSK1 (#9591), phospho‐Ser133 CREB (#9198), GAPDH (#2118), phospho‐Ser376 MSK1 (#9591), phospho‐Ser82 Hsp27 (#2406), phospho‐Thr334 MK2 (#3041) and total MK2 (#3042) were from Cell Signaling Technology (Hitchin, UK) and used at a dilution of 1 in 1000. Sheep anti‐PKB alpha (S742B) and sheep anti‐MSK1 (S804B) were from the Division of Signal Transduction Therapy (University of Dundee) and used at a concentration of 1 μg mL^−1^. The phospho‐Ser92 Brf1 antibody was from Abcam (Cambridge, UK, #AB79191) and used at 1:1000. Typically, two biological replicates were included in blotting experiments and data shown are from one replicate. The experiments shown are representative of multiple experiments.

### 14‐3‐3 pulldown

BMMCs were pretreated for 1 h with DMSO or 5 μm Cmp2s before stimulation with 10 ng mL^−1^ IL‐33 (PeproTech). Cells were lysed in 50 mm Tris‐HCl (pH 7.5), 1 mm EGTA, 1 mm EDTA, 1 mm sodium orthovanadate, 50 mm sodium fluoride, 1 mm sodium pyrophosphate, 0.27 m sucrose, 1% (vol/vol) Triton X‐100, 0.1% (vol/vol) 2‐mercaptoethanol, 1 μg mL^−1^ aprotinin, 1 μg mL^−1^ leupeptin, 1 mm PMSF (triton lysis buffer). Lysates were clarified by centrifugation (13,000 rpm for 10 min at 4°C). Protein concentration was determined with Coomassie Protein Assay Reagent. A quantity of 1 mg of lysate per condition was precleared using Sepharose beads for 1 h and then incubated for 16 h at 4°C with 14‐3‐3‐Sepharose beads.^70^ Beads were pelleted by centrifugation and washed two times in triton lysis buffer with 0.15 m NaCl and once in triton lysis buffer. When indicated in the figure legends, samples were treated with phosphatase to dephosphorylate the proteins. To phosphatase treat samples, following the pulldown beads were pelleted by centrifugation and washed three times in PMP buffer (New England Biolabs, Hitchin, UK) and treated with or without 1000 units of λ phosphatase (New England Biolabs) in PMP buffer at 30°C for 30 min. 2X SDS sample buffer was added directly to beads which were then boiled for 10 min to release bound proteins.

### qPCR analysis

Total RNA was extracted from cells following stimulation using RNeasy kits (Qiagen, Manchester, UK), according to manufacturer's instructions. A quantity of 0.5–1 μg RNA was reverse transcribed into cDNA using iScript and the resulting cDNA analyzed by qPCR using a Sybr‐Green based detection (Takara Bio Europe, Saint‐Germain‐en‐Laye, France). 18s and/or GAPDH were used for normalization and results calculated as fold stimulation relative to the unstimulated wild‐type cells as described.^42^ Primers used are listed in Supplementary table 2.

### Analysis of cytokine levels

Following stimulation of cells, the levels of TNF, IL‐6, IL‐10, IL‐13, IL‐12p40, GM‐CSF, CCL3, CCL4 and CXCL2 present in the media were determined via a multiplex Luminex based method (Bioplex, BioRad, Watford, UK). For the analysis of intracellular TNF levels, BMMCs were stimulated for 4 h with 10 ng mL^−1^ IL‐33 in the presence of 3 μg mL^−1^ Brefeldin A and 2 μm Monensin to block cytokine secretion. Cells were then incubated with Fc Block (1 in 50 dilution, BD Biosciences) for 10 min, fixed and permeabilized (Fixation/Permeabilization Kit, BD Biosciences) and stained for TNF (TNF‐PE, clone TN3‐19.12, Biolegend, 1 in 200) and analyzed by flow cytometry.

### Peritoneal neutrophil recruitment model

Mice were injected intraperitoneally with 100 μL PBS, or 5 μg kg^−1^ IL‐33 (PeproTech) or 2.5 mg kg^−1^ LPS (*Escherichia coli* strain O26:B6; Sigma; L2654) in 100 μL PBS. After 3 h, the mice were sacrificed via an increasing concentration of CO_2_, and the peritoneal cavity was washed using 4 mL PBS containing 1% BSA and 5 mm EDTA. Cells were pelleted by centrifugation and incubated on ice with Fc Block (1 in 50 dilution, BD Biosciences) for 10 min. Cells were then stained with anti‐Gr1‐PerCp‐Cy5.5 (1 in 800, BD Biosciences, #552093) and anti‐CD11b‐APC (1 in 1600, BD Biosciences #553312) for 30 min. Cells were then washed twice and analyzed by flow cytometry. For analysis, live cells were gated based on a forward and side scatter and neutrophils defined as CD11b Gr1 double‐positive cells. Gating and representative FACS plots are shown in Supplementary figure 12.

### Neutrophil isolation

Mouse bone marrow‐derived neutrophils were isolated from femurs and tibias as previously described.^71^ Briefly, bone marrow was flushed from the bones with ice cold PBS and separated on a gradient prepared by overlaying 3 mL Histopaque 1119 (Sigma), 3 mL Histopaque 1077 (Sigma‐Aldrich, Gillingham, UK) and 1 mL PBS containing the bone marrow cells. The gradient was centrifuged for 30 min at 900 *g* without brakes, and neutrophils were collected from the interface of Histopaque 1119 and Histopaque 1077.

### *In vitro *chemotaxis assay

BMMCs (1 x 10^6^ cells) in 600 μL mast cell media in a 24‐well plate were stimulated with 10 ng mL^−1^ IL‐33 for 4 h or left unstimulated. ThinCert Transwell inserts with 3 μm pore size (Greiner bio‐one) were allowed to equilibrate in wells for 10 min. Neutrophils (1 x 10^6^) in 200 μL mast cell media were placed in the upper compartment of each of the transwell chambers. After incubation for 1 h at 37°C and 5% CO_2_, cells were harvested from both upper and lower chambers. GR‐1^+^ CD11b^+^ were enumerated using flow cytometry by comparison with Precision Count beads (BioLegend) added to each sample. Neutrophil migration was determined as the percentage of neutrophils migrating to the lower chamber as a percentage of total neutrophils.

### Statistical analysis

Data are presented as mean values ± s.d. unless otherwise stated. A Student's *t*‐test (two‐tailed, unpaired) was performed in Excel and ANOVA testing in SigmaPlot. For post hoc analysis of ANOVA tests, the Holm–Sidak method was used.

## Conflict of Interest

KP is employed by AstraZeneca, and owns shares in the company.
