# Supplementary material for: IL‐33 regulates cytokine production and neutrophil recruitment via the p38 MAPK‐activated kinases MK2/3
Source: Immunol Cell Biol. 2018 Oct 19;97(1):54–71. doi: 10.1111/imcb.12200 (PMC6378613; doi:10.1111/imcb.12200)
Supplement: Supplementary file 1 [file IMCB-97-54-s001.pdf]

Supplementary figures:

IL-33 regulates cytokine production and neutrophil recruitment via the p38 activated kinases MK2/3.

Pierre C. McCarthy[1,2*], Iain R. Phair[1*], Corinna Greger[1], Katerina Pardali[3], Victoria A. McGuire[1,4] Andrew R. Clark[5], Matthias Gaestel[6] and J. Simon C. Arthur[1+].

1 Division of Cell Signalling and Immunology, School of Life Sciences, Wellcome Trust Building, University of Dundee, Dow St, Dundee, DD1 5EH, UK.

2 MRC Protein Phosphorylation Unit, School of Life Sciences, Sir James Black Centre, University of Dundee, Dow St, Dundee, DD1 5EH, UK.

3 R&I iMed, Translational Science, AstraZeneca, Pepparedsleden 1, 431 83 Mölndal, Sweden.

4 Scottish Cutaneous Porphyria Service, Photobiology Unit, Ninewells Hospital and Medical School, Dundee, DD1 9SY, UK.

5 Institute of Inflammation and Ageing, College of Medical and Dental Sciences, University of Birmingham, Birmingham, B15 2TT, UK.

6 Institute for Cell Biochemistry, Hannover Medical School, Carl-Neuberg-Str. 1, Hannover 30623, Germany.

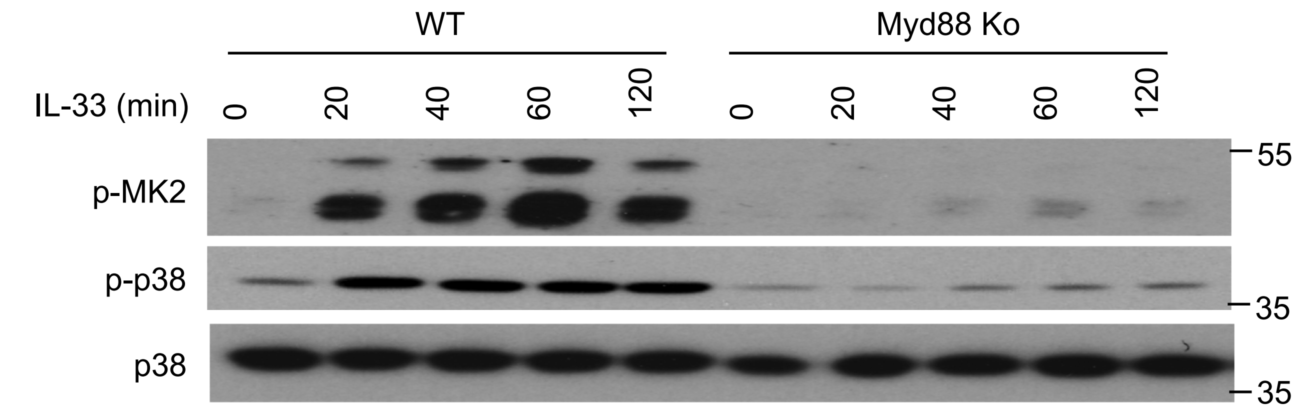

Supplementary Figure 1. IL-33 signals via Myd88.
BMMCs were isolated from wild type or Myd88 knockout mice and stimulated for the indicated times with 10 ng mL^{-1} IL-33. Cells were lysed and the levels of phospho MK2, phospho p38 and total p38 determined by immunoblotting.

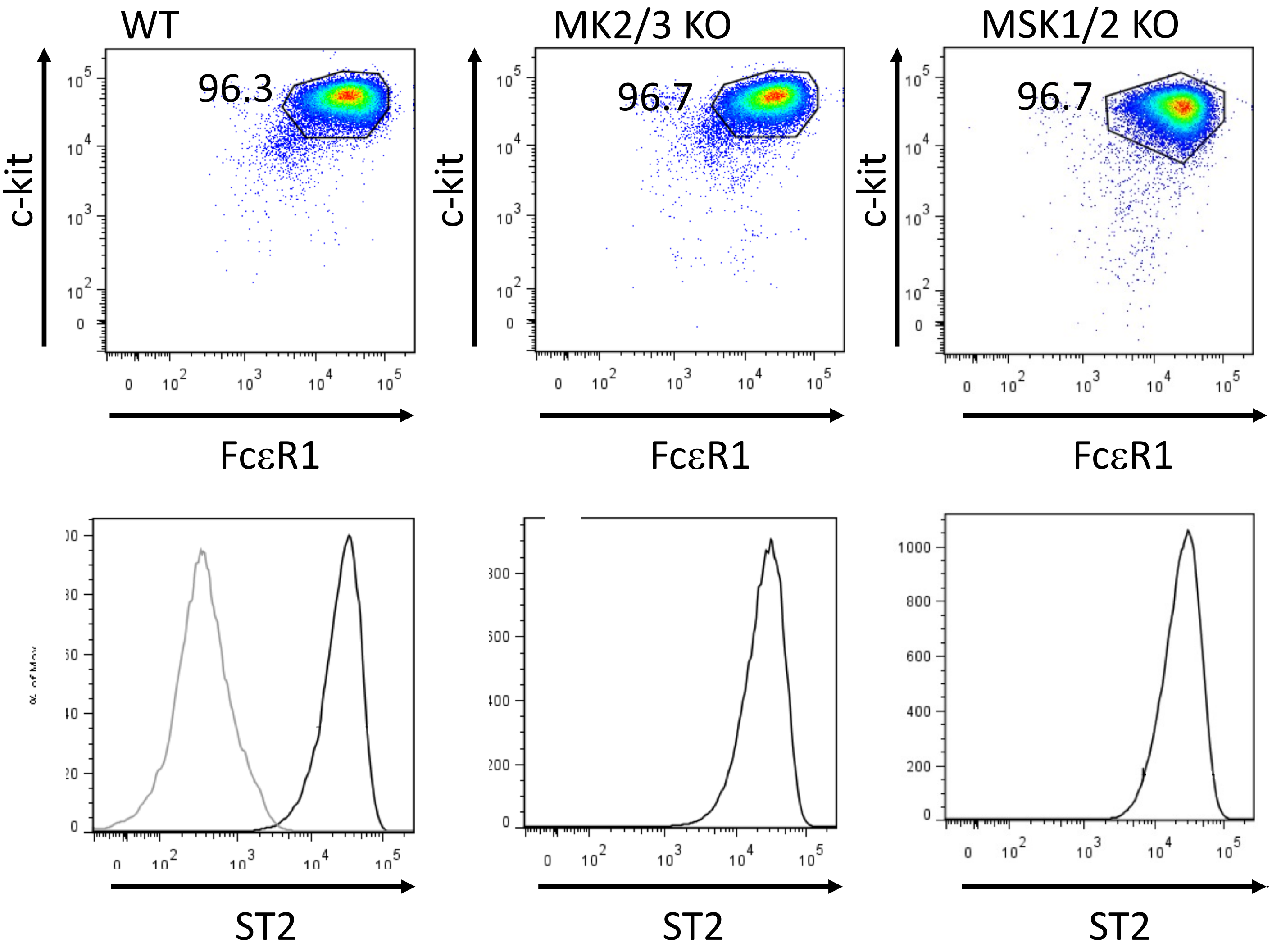

Supplementary Figure 2. Knockout of MK2/3 or MSK1/2 does not affect BMMC differentiation of ST2 expression.
BMMCs were isolated from wild type, MK2/3 knockout or MSK1/2 knockout mice. Cell surface levels of c-kit and FcεR1 and ST2 were determined by flow cytometry. Levels of c-kit and FcεR1 are shown in the top panel and the level of ST2 in the c-kit/FcER1 positive gate shown in the bottom panel. For the WT cells, the grey line in the histogram indicates background fluorescence i.e. cells without the ST2 antibody.

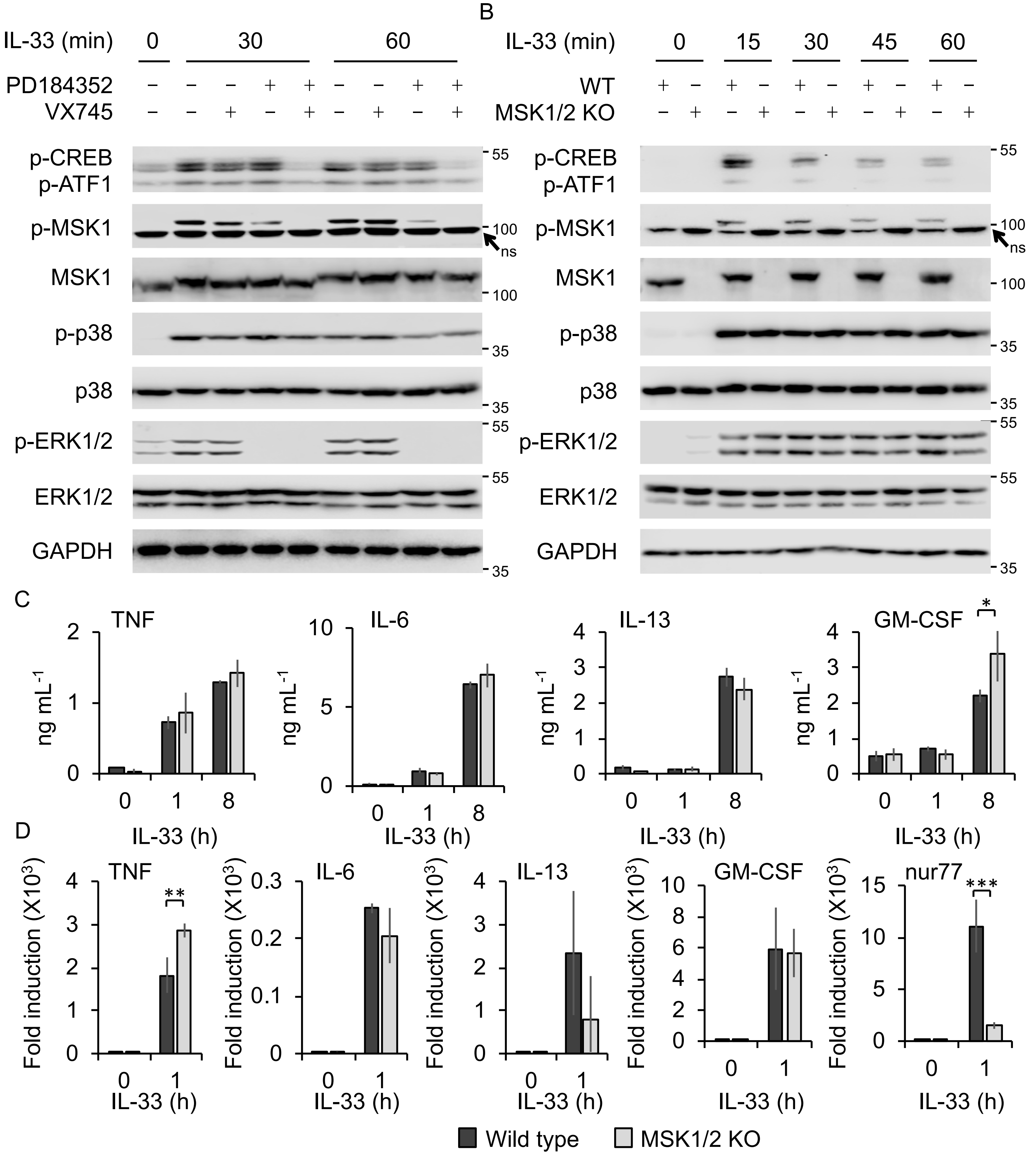

Supplementary Figure 3 Effect of MSK1/2 knockout on IL-33 induced cytokine production in BMMCs

(A) BMMCs from wild type mice were pre-incubated with 2 μM PD184352 and/or 1 μM VX745 as indicated and then stimulated for the times shown with 10 ng mL^{-1} IL-33. The levels of the indicated total and phosphorylated proteins was determined by immunoblotting. ns = non-specific band.

(B) As (A) but cells were isolated from wild type of MSK1/2 knockout mice.

(C) BMMCs from wild type or MSK1/2 knockout mice were stimulated with 10 ng mL^{-1} IL-33 for the indicated times. The levels of TNF, IL-6, IL-13 and GM-CSF in the media at the indicated times were then determined.

(D) As (C) but cells were lysed after 1h, total RNA extracted and the induction of the indicated genes determined by qPCR as described in the methods. Graphs show the mean and standard deviation of independent cultures from 4 mice per genotype. A *P* value (two tailed Students *t*-test) between wild type and knockout of less than 0.05 is indicated *, less than 0.01 by ** and less than 0.001 by ***.

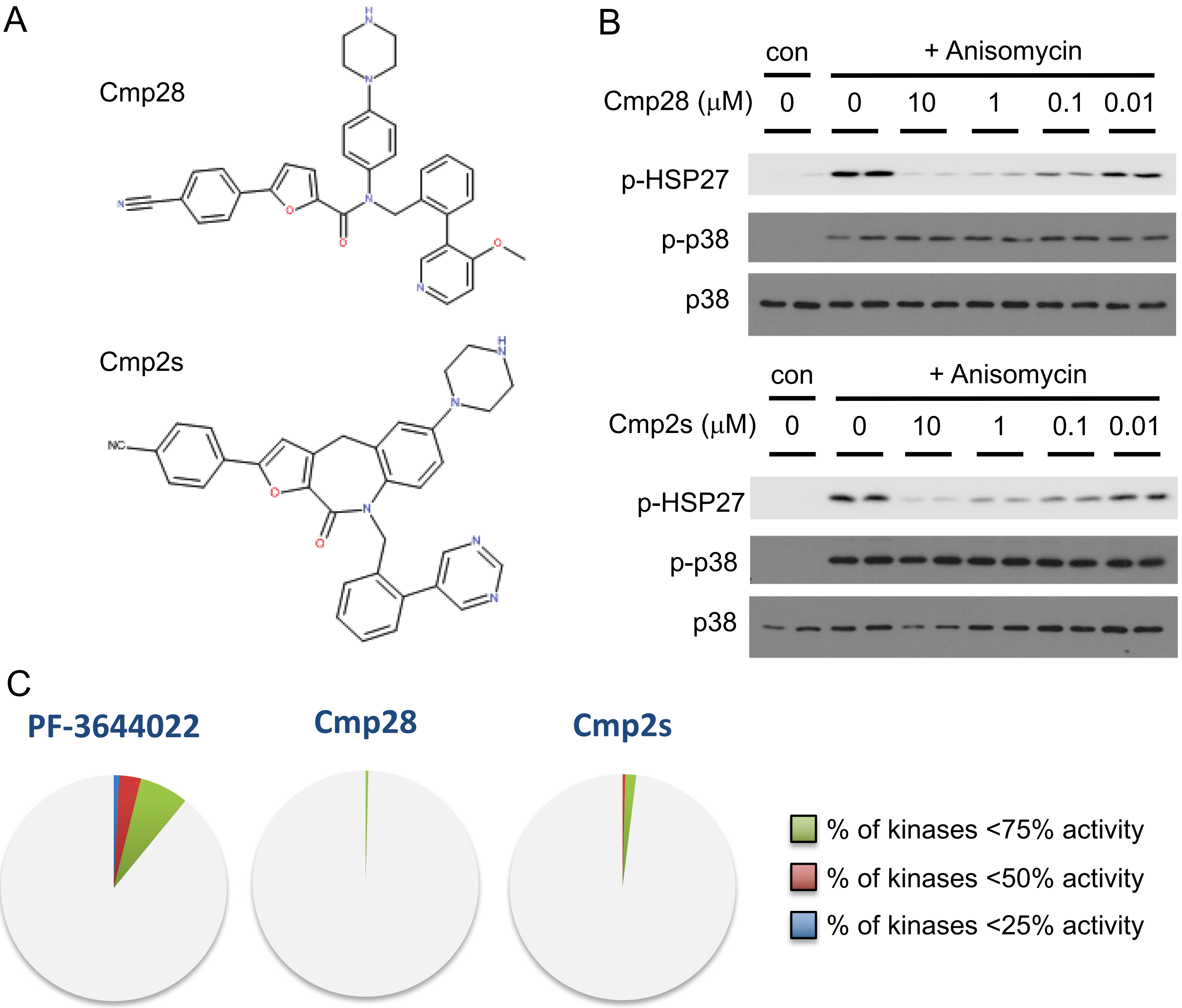

Supplementary Figure 4. Characterisation of MK2 inhibitors.

A) Structures of Cmp28 and Cmp2s.

B) HeLa cells were pre-treated for 1h with the indicated concentrations of Cmp28 or Cmp2 and then stimulated with 10 μg mL-1 anisomycin for 30 min. The levels of phospho Hsp27, phospho p38 and total p38 were determined by immunoblotting.

C) PF-3644022 was screened against a panel of 129 kinases while Cmp28 and Cmp2 were screened against a panel of 291 wild type or mutated kinases, containing 256 kinases genes. All inhibitors were screened at 1 μM and charts represent the % of of target hits from the 129 or 256 kinases inhibited by 25, 50 or 75% in the assays. A full list of the results is shown in supplementary table 1.

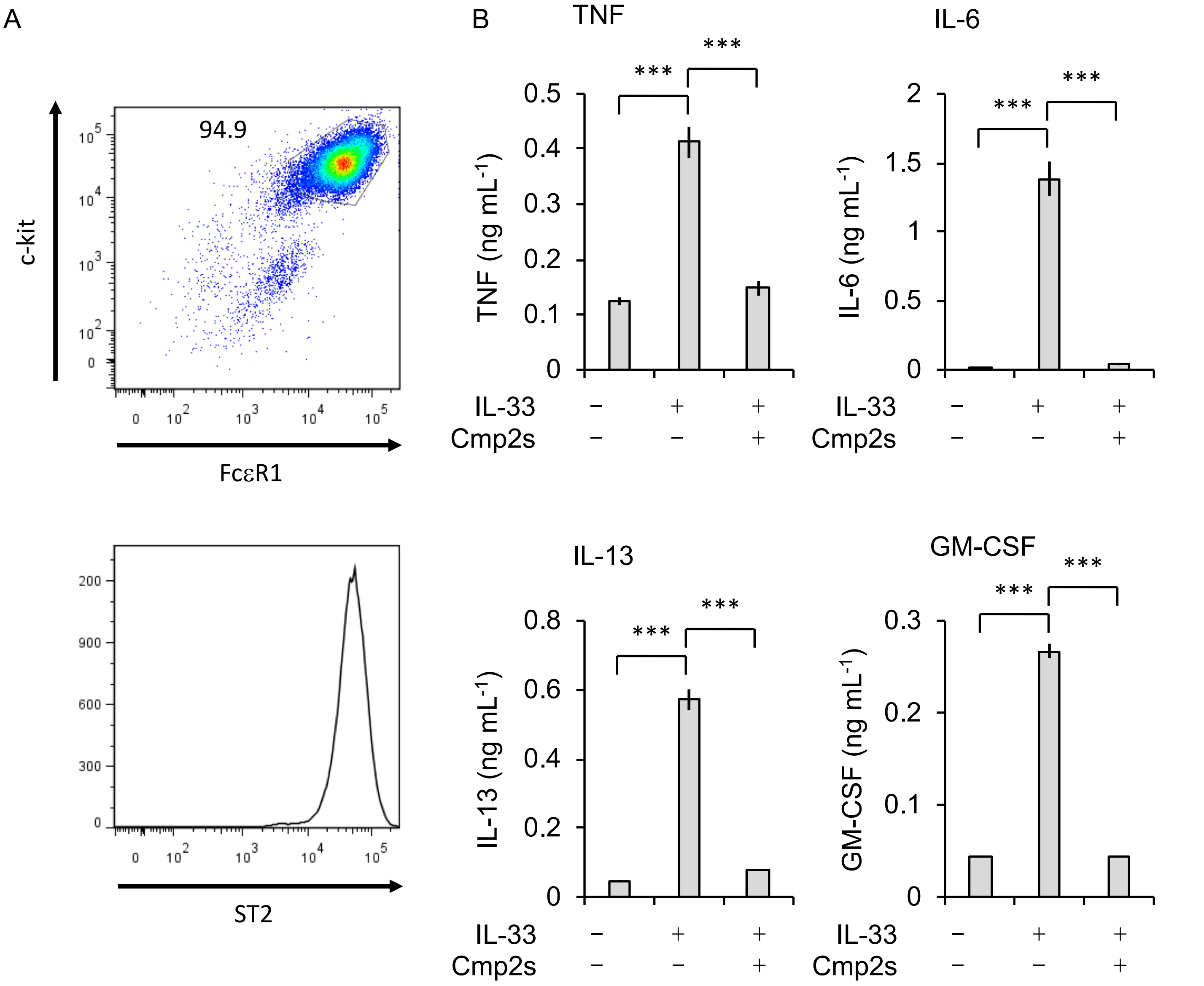

Supplementary Figure 5. Effect of MK2 inhibition on peritoneal mast cells.
Peritoneal mast cells were cultured as described in the methods. Cell purity prior to stimulation was assessed by flow cytometry. Plots show c-kit and FcεR1 expression on live cells (gated on forward and side scatter) and ST2 expression in c-kit and FcεR1 positive cells (A). Where indicated cells were stimulated with 5 μM Cmp2s before stimulation with 10ng mL^{-1} IL-33 for 8h. Secreted levels of TNF, IL-6, IL-13 and GM-CSF were measured by a Luminex based multiplex assay (B). Graphs show the mean and standard deviation of 4 stimulations per condition. A *P* value for comparisons to the IL-33 stimulated condition of less than 0.001 (two tailed Students *t-test*) is indicated by ***.

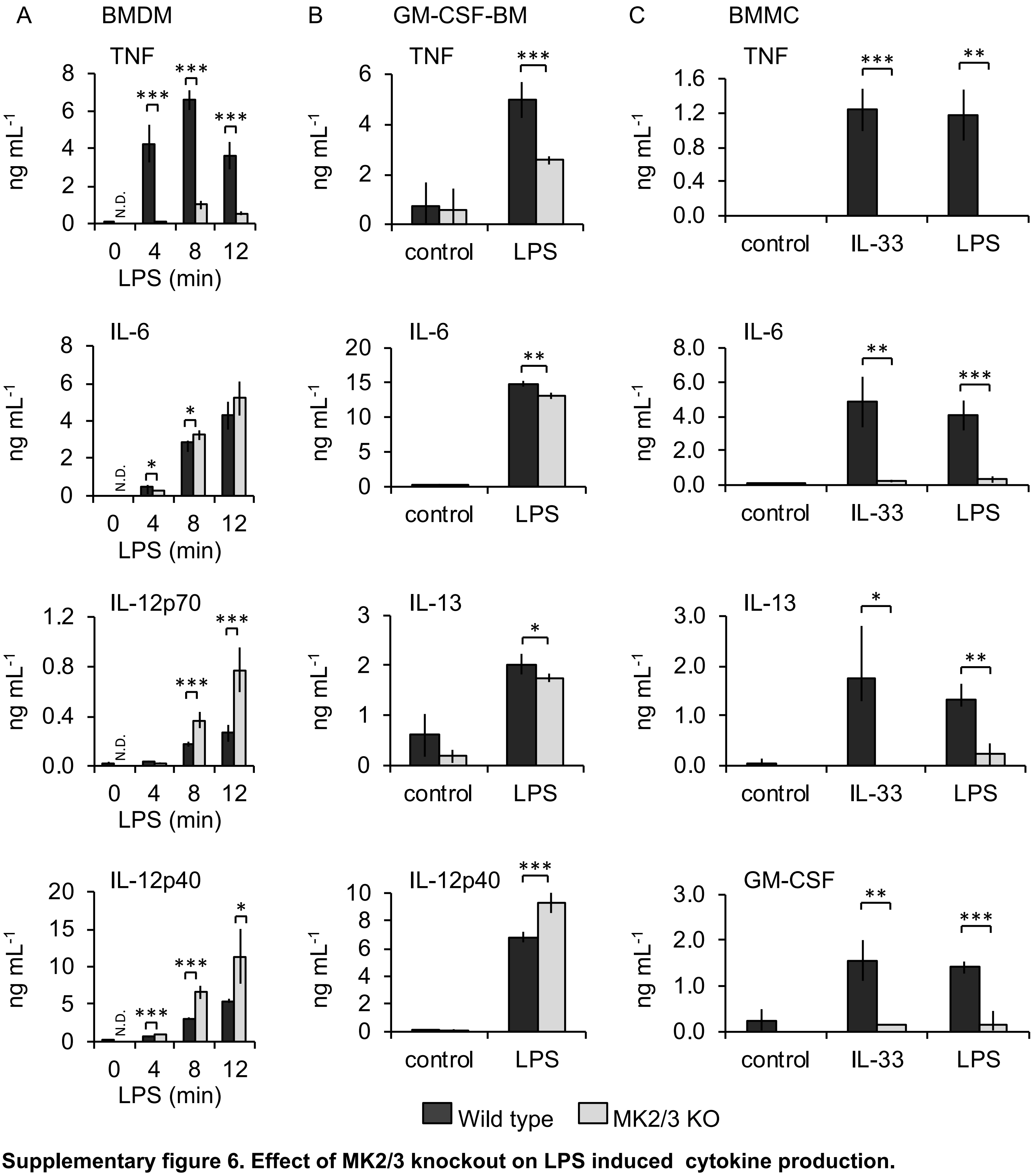

Supplementary figure 6. Effect of MK2/3 knockout on LPS induced cytokine production.
A) Bone marrow from wild type or MK2/3 knockout mice was differentiated for 7 days in culture with M-CSF. Cells were then split and stimulated on day 8 for 8 h with 100 ng mL^{-1} LPS for the times indicated. The levels of the indicated cytokines secreted into the media was determined.
B) As (A) but bone marrow from wild type or MK2/3 knockout mice was differentiated for 7 days in culture with GM-CSF. Secreted cytokines were analysed after 16h of stimulation with 100 ng mL^{-1} LPS
C) BMMCs were stimulated with either 10ng mL^{-1} IL-33 or 100 ng mL^{-1} LPS for 8h and the levels of TNF, IL-6, IL-13 and GM-CSF secreted into the media was determined. In A-C graphs show the mean and standard deviation of independent cultures from 4 mice per genotype except for (C) where 3 independent MK2/3 cultures were used. A *P* value (two tailed Students *t*-test) between wild type and knockout of less than 0.05 is indicated *, less than 0.01 by ** and less than 0.001 by ***.

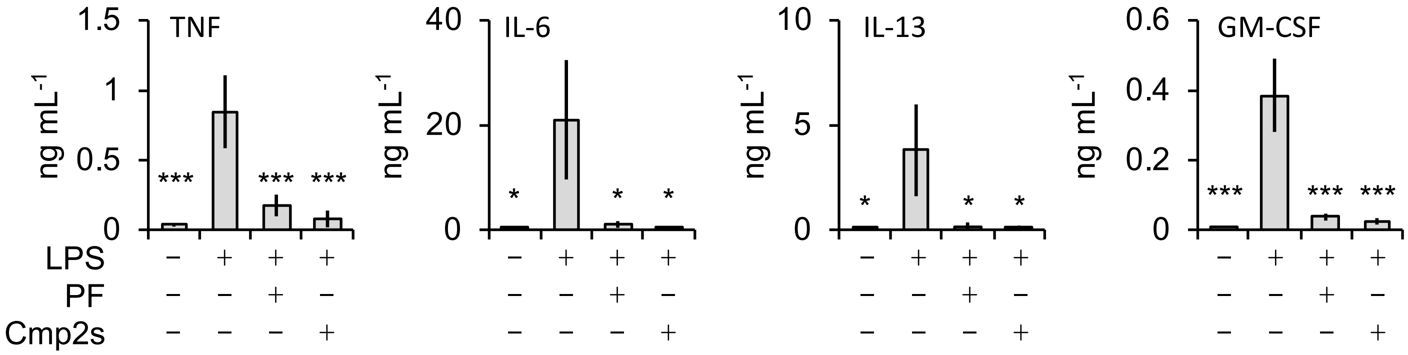

Supplementary Figure 7. Effect of MK2 inhibitors on LPS stimulated cytokine production
BMMCs were isolated from wild type mice and where indicated pretreated for 1h with 5 μM PF-3644022 or 5 μM Cmp2s. Cells were then stimulated with 100 ng mL^{-1} LPS for 8h and levels of the indicated cytokines in the media determined. Graphs show mean and standard deviation of results from cultures from 3 mice. For comparisons to the LPS stimulated condition $P < 0.05$ in indicated by * and $P < 0.001$ by ** (post hoc Holm Sidak test following one way ANOVA).

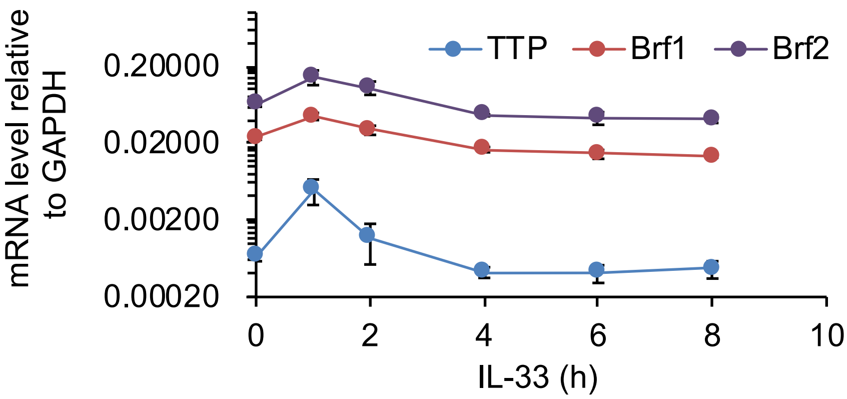

Supplementary Figure 8. Effect of IL-33 on Brf mRNA levels.
BMMCs were isolated from wild type mice and stimulated for the indicated times with 10 ng mL-1 IL-33. Cells were lysed and the levels of Brf1, Brf2 and TTP mRNA levels relative to the level of GAPDH mRNA determined by qPCR. Graphs show mean and standard deviation of results from cultures from 4 mice.

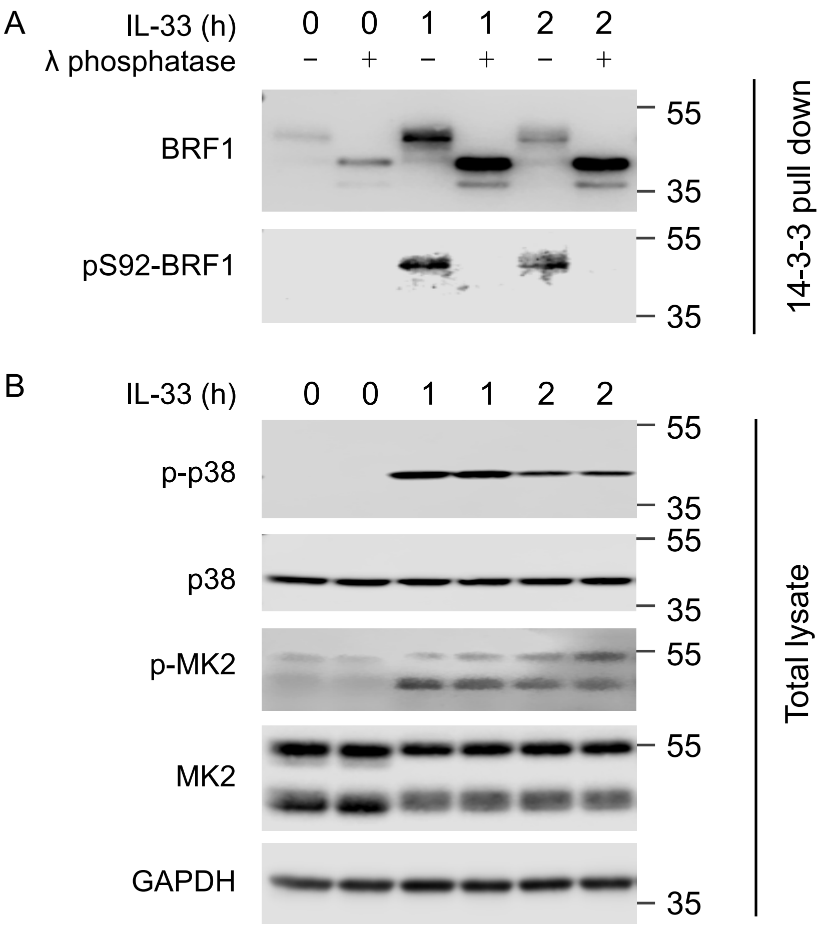

Supplementary Figure 9. Phosphatase treatment of 14-3-3 pulldowns.
BMMCs were isolated from wild type mice and stimulated for the indicated times with 10 ng mL^{-1} IL-33. Cells were lysed 14-3-3 pull downs performed on the soluble fraction of the cell lysate as described in the methods. Pull downs were then left untreated or treated with 1000U of lambda phosphatase. Samples were then run on polyacrylamide gels and blotted for total Brf1 or phospho S92 Brf1 (A). Alternatively samples of the cell lysates were blotted for phospho and total p38, phospho and total MK2 or GAPDH (B).

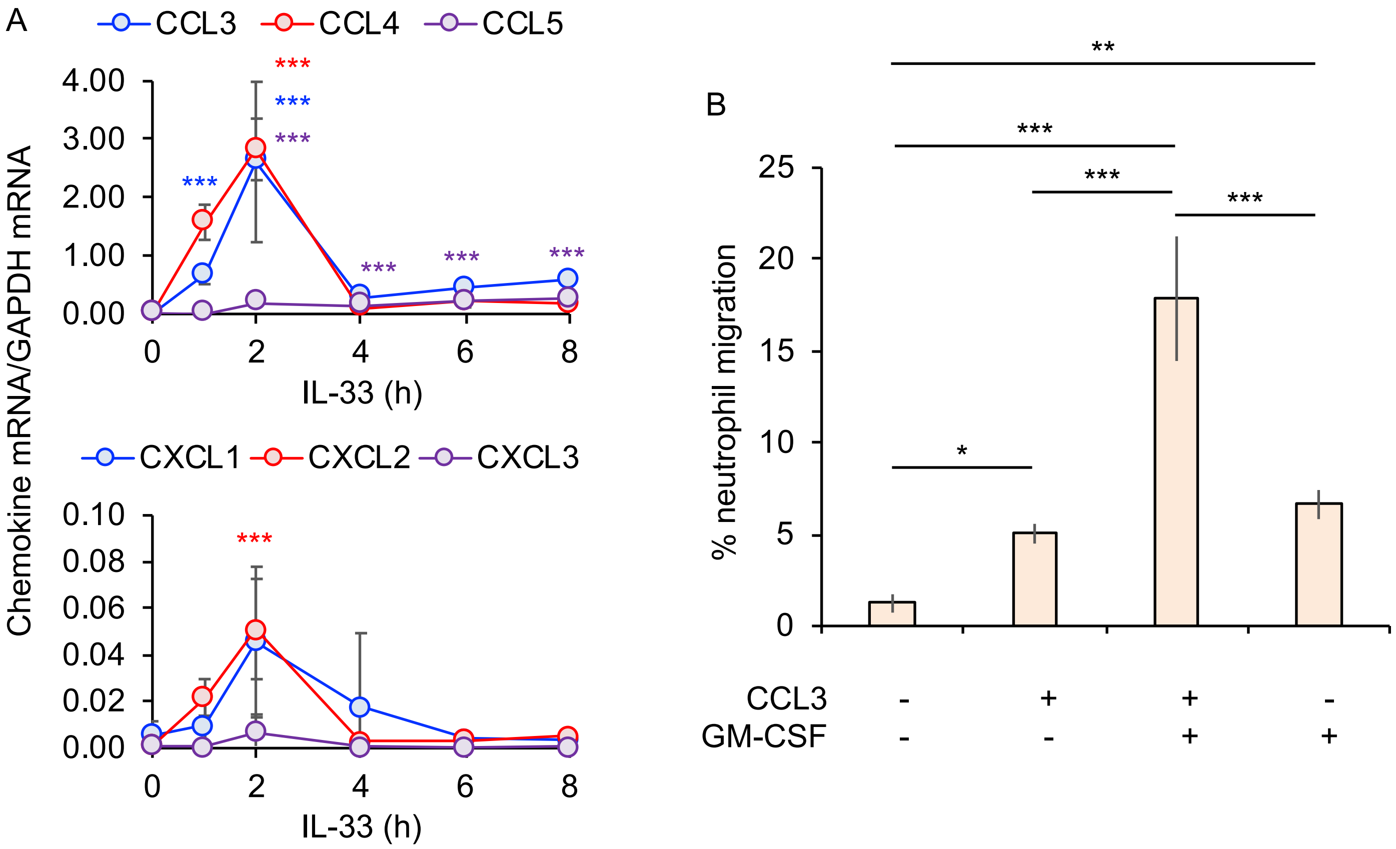

Supplementary Figure 10. Chemokine mRNA induction in BMMCs.
A) BMMCs were treated with 10 ng mL^{-1} IL-33 for the indicated times. Total RNA was isolated and levels of CXCL1, CXCL2, CXCL3, CCL3, CCL4, CCL5 and GAPDH determined by qPCR. Levels of the chemokines relative to GAPDH were the calculated. Results show mean and standard deviation of 4 biological replicates. One way ANOVA showed a significant effect of IL-33 for CXCL1 (F=2.79, P=.049), CXCL2 (F=17.409, $P < 0.001$), CCL3 (F=10.77, $P < 0.001$), CCL4 (F=79.26, $P < 0.001$) and CCL5 (F=40.21, $P < 0.001$). For post hoc analysis (Holm Sidak method) of time points relative to the 0 time point $P < 0.001$ is indicated by ***. B) The migration of bone marrow neutrophils was measured over 1 h in Transwell assays as described in the methods. The neutrophils were placed in the top chamber and the lower chamber contained media with 1 ng mL^{-1} CCL3 or 10 ng mL^{-1} GM-CSF as indicated. One way ANOVA showed a significant effect treatment (F=64.243, $P < 0.001$). For post hoc analysis (Holm Sidak method) $P < 0.05$ is indicated by *, < 0.01 by ** and < 0.001 by ***.

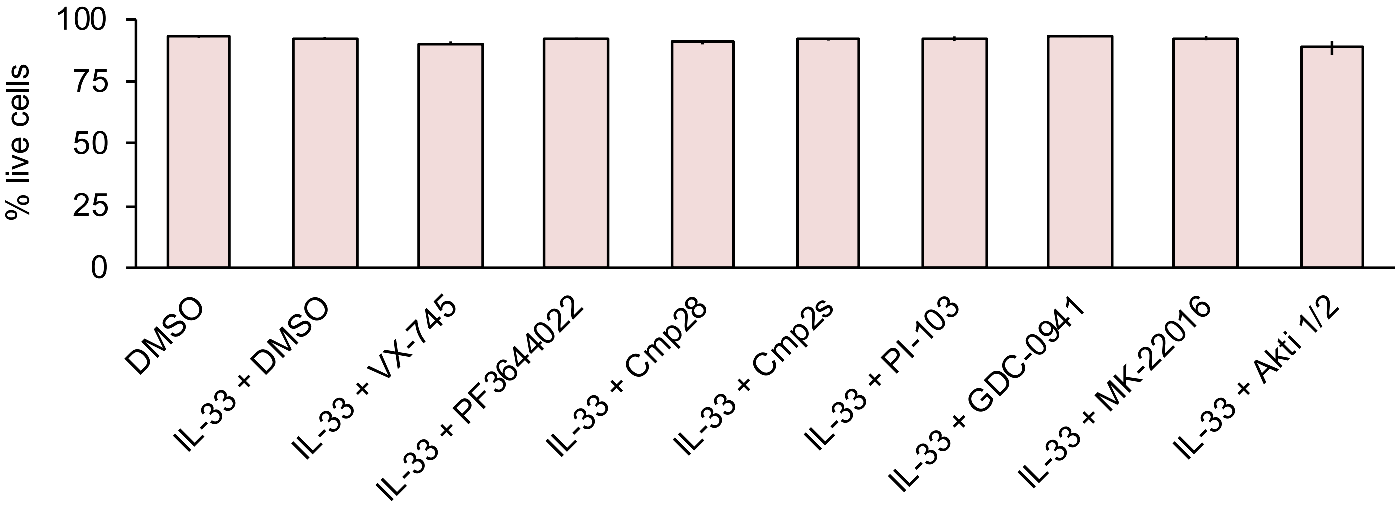

Supplementary Figure 11. Viability of mast cells in the presence of kinase inhibitors.
BMMCs were treated with DMSO (vehicle control) or the indicated inhibitors at the concentrations listed in the methods. After 1h cells were stimulated for 16h with 10 ng mL-1 IL-33 or left unstimulated. Cells were then stained for FcεR (to confirm mast cell identity) and DAPI (to determine viability) then analysed by flow cytometry. The % of viable FcεR+ve cells based on DAPI staining is shown. Results show mean and standard deviation, n=3.

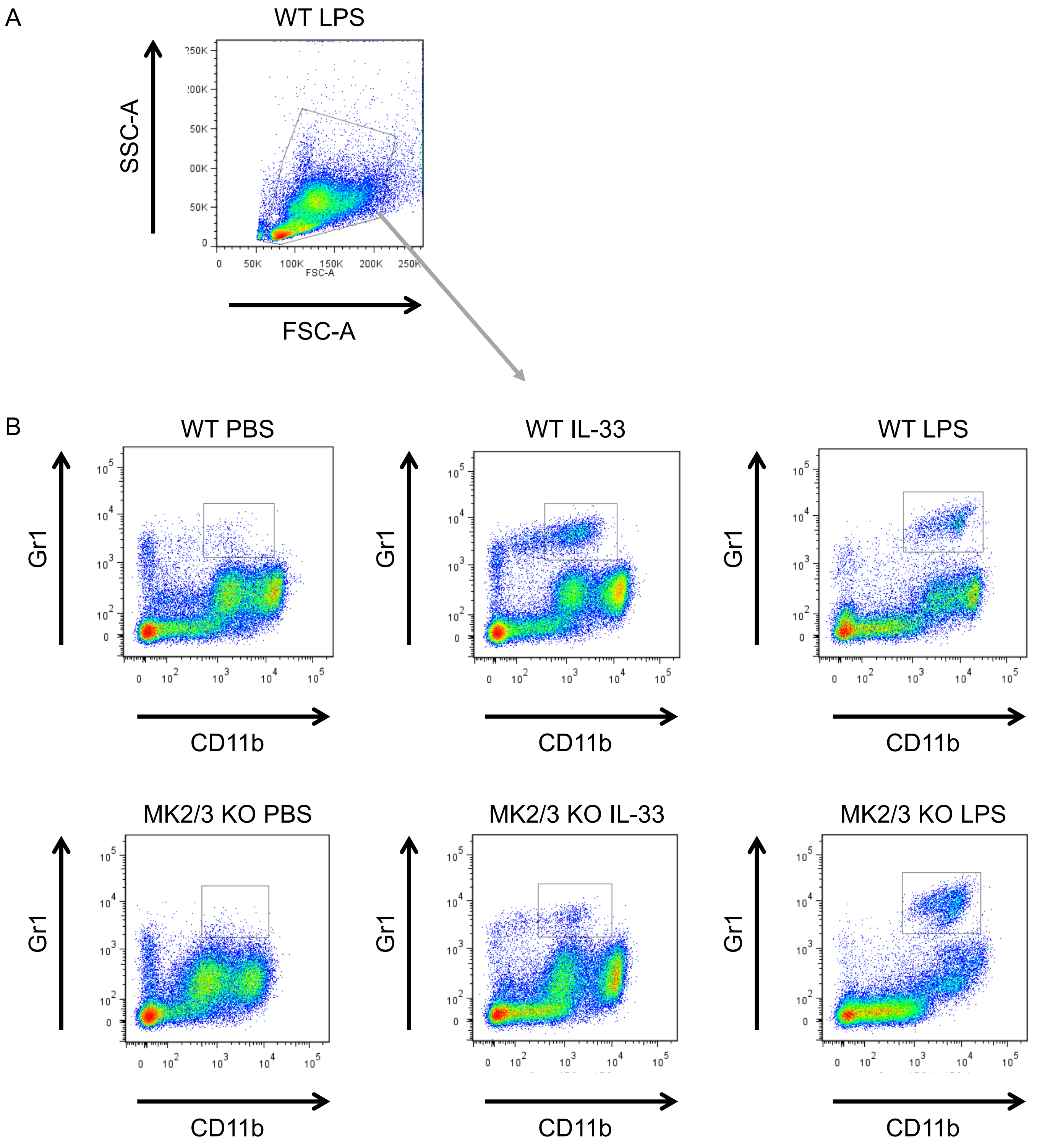

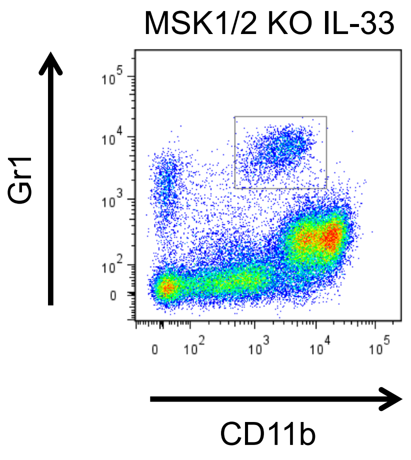

Supplemental Figure 12. IL-33 induced neutrophil recruitment
Wild type, MK2/3 or MSK1/2 were injected with either PBS, 5 µg/kg IL-33 or 2 mg/kg LPS. 3 h later mice were sacrificed and the peritoneum washed to obtain cells. The % of neutrophils (CD11b+ve / Gr1+ve) in the peritoneal wash was determined by staining for CD11b and Gr1 and analysed by flow cytometry. Live cells were first selected based on forward / side scatter (representative example shown in A). Neutrophils (grey boxes) were defined as CD11b+ve Gr1+ve cells. Representative plots from the mice used in Figure 7 are shown in (B)
