# Supplementary material for: IL‐33 regulates cytokine production and neutrophil recruitment via the p38 MAPK‐activated kinases MK2/3
Source: Immunol Cell Biol. 2018 Oct 19;97(1):54–71. doi: 10.1111/imcb.12200 (PMC6378613; doi:10.1111/imcb.12200)
Supplement: Supplementary file 3 [file IMCB-97-54-s003.pdf]

Supplementary table 2 Primer sequences used for qPCR

Gene	Sense	Antisense
IL-6	TTCCATCCAGTTGCCTTCTTG	AGGTCTGTTGGGAGTGGTATC
IL-6 primary transcript	TGCTATCTGCTCACTTGCCG	CACCAGCATCAGTCCCAAGA
IL-13	GCAGCAGCTTGAGCACATTT	GCAGACAGGAGTGTTGCTCT
IL-13 primary transcript	AGGAGCTGAGCAACATCACA	CCTGCTCATGACCTCTTCCG
GM-CSF	CTCACCCATCACTGTCACCC	TGAAATTGCCCCGTAGACCC
GM-CSF primary transcript	GGAGCAGAAGGGAGTAGGGA	AAATTGCCCCGTAGACCCTG
TNFa	CAGACCCTCACACTCAGATCATC	GGCTACAGGCTTGTCACTCG
TNFa primary transcript	CCGTGGGTTGGACAGATGAA	TGAGGGTCTGGGCCATAGAA
Nur77	CCTGTTGCTAGAGTCTGCCTTC	CAATCCAATCACCAAAGCCACG
CCL3	AAGGATACAAGCAGCAGCGA	CGTGGAATCTTCCGGCTGTA
CCL4	CCCAGCTCTGTGCAAACCTA	CCATTGGTGCTGAGAACCCT
CCL5	TGCTCCAATCTTGCAGTCGT	GCAAGCAATGACAGGGAAGC
CXCL1	TGGCTGGGATTCACCTCAAG	CCGTTACTTGGGGACACCTT
CXCL2	AGGGCGGTCAAAAAGTTTGC	CAGGTACGATCCAGGCTTCC
CXCL3	GAAAGGAGGAAGCCCCTCAC	AACAAGCAGGTAAAGACACATCC
GAPDH	TGCACCACCAACTGCTTAG	GATGCAGGGATGATGTTC
18S	GTAACCCGTTGAACCCCATT	CCATCCAATCGGTAGTAGCG
